# Macrovascular contributions to resting-state fMRI signals: A comparison between EPI and bSSFP at 9.4 Tesla

**DOI:** 10.1162/imag_a_00435

**Published:** 2025-01-07

**Authors:** Dana Ramadan, Sebastian Mueller, Ruediger Stirnberg, Dario Bosch, Philipp Ehses, Klaus Scheffler, Jonas Bause

**Affiliations:** High-Field Magnetic Resonance Center, Max Planck Institute for Biological Cybernetics, Tuebingen, Germany; German Center for Neurodegenerative Diseases (DZNE), Bonn, Germany; Department of Biomedical Magnetic Resonance, University of Tuebingen, Tuebingen, Germany

**Keywords:** resting-state fMRI, cortical orientation, layer fMRI, macrovascular contributions, GRE-EPI, bSSFP

## Abstract

The draining-vein bias of T2^*^-weighted sequences, like gradient echo echo-planar imaging (GRE-EPI), can limit the spatial specificity of functional MRI (fMRI). The underlying extravascular signal changes increase with field strength (B_0_) and the perpendicularity of draining veins to the main axis of B_0_, and are, therefore, particularly problematic at ultra-high field (UHF). In contrast, simulations showed that T2-weighted sequences are less affected by the draining-vein bias, depending on the amount of rephasing of extravascular signal. As large pial veins on the cortical surface follow the cortical folding tightly, their orientation can be approximated by the cortical orientation to$\vec{B_{0}}$. In our work, we compare the influence of the cortical orientation to$\vec{B_{0}}$on the resting-state fMRI signal of three sequences aiming to understand their macrovascular contribution. While 2D GRE-EPI and 3D GRE-EPI (both T2^*^-weighted) showed a high dependence on the cortical orientation to$\vec{B_{0}}$, especially on the cortical surface, this was not the case for 3D balanced steady-state free precession (bSSFP) (T2/T1-weighted). Here, a slight increase of orientation dependence was shown in depths closest to white matter (WM). And while orientation dependence decreased with increased distance to the veins for both EPI sequences, no change in orientation dependence was observed in bSSFP. This indicates the low macrovascular contribution to the bSSFP signal, making it a promising sequence for layer fMRI at UHF.

## 1 Introduction

The carotid and vertebral arteries supply blood to the brain, branching into large arteries on the cortical surface, also called pial arteries. Diving intracortical arteries are oriented perpendicular to the cortical surface and large pial arteries. These diving vessels branch into randomly oriented arterioles (Duvernoy et al., 1981) and capillaries, where essentially gas exchange and nutrient delivery occur for glia cells and neurons. The capillary branches connect to the venous blood stream through venules (∼10μm), which merge into intracortical ascending veins (∼80μm) oriented perpendicular to the large veins (≥200μm) on the cortical surface. The latter are referred to as pial or draining veins, as they drain the blood to the large cerebral veins. This means that both the arterial and venous vessels of the cortex follow the curvature closely. For this reason, the cortical orientation with respect to the main magnetic field$\vec{B_{0}}$is a valid estimate for the orientation of the pial vasculature to$\vec{B_{0}}$.

It is known that the brain lacks an energy reservoir of its own, so blood flow is essential to supply the brain with the required energy on demand. When neurons are active, blood flow typically increases in their vicinity, and strong blood flow from activated larger areas accumulates in more distant larger veins. This phenomenon is referred to as neurovascular coupling (Attwell et al., 2010;Iadecola, 2004,^2017^). For unknown reasons, active brain regions are flooded with more oxygenated blood than required (Raichle & Mintun, 2006). With this change in blood flow, blood volume, and oxygenation level, also known as the hemodynamic response, the blood oxygenation level-dependent (BOLD) effect arises (Ogawa et al., 1990), which is employed in the majority of functional magnetic resonance imaging (fMRI) experiments.

The deoxyhemoglobin in venous blood is paramagnetic and, therefore, perturbs the magnetic field in the surrounding diamagnetic tissue (Pauling & Coryell, 1936). If veins are modeled as infinitely long cylinders filled with paramagnetic material, the field offsets surrounding them are highest, when perpendicular to the main axis of$\vec{B_{0}}$(Chu et al., 1990). Using this long cylinder simplification, the following equations can be used to describe the extravascular ($\Delta \omega_{extra}$), as well as the intravascular ($\Delta \omega_{intra}$) frequency offsets resulting from susceptibility differences between oxygenated and deoxygenated blood (Δχ):

$$\begin{array}{cc} \Delta \omega_{extra}\propto & \Delta \chi \omega_{0}{\left(\frac{R}{r}\right)}^{2}cos\left(2\phi \right)sin^{2}(\nu ) \end{array}$$ (1)

$$\Delta \omega_{intra}\propto \;\;\;\;\;\;\;\;\;\;\;\;\;\;\;\Delta \chi \omega_{0}\left(3cos^{2}(\nu )-1\right).$$ (2)

Here,νis the angle between$\vec{B_{0}}$and the main axis of the cylinder,Rits radius,rthe distance between any observation point and the axis of the cylinder (r≥R), andϕthe polar angle of the position vector$\vec{r}$in the plane perpendicular to the cylinder axis. The Larmor frequency$\omega_{0}$is directly proportional to B_0_, thus extra- and intravascular frequency offsets are stronger at higher field strengths. Since$\Delta \omega_{extra}$inEquation 1depends on the ratio$\frac{R}{r}$, vessels with larger radius cause a more long-range field distortion than vessels with smaller radius. Due to larger spatial extent of this effect, the extravascular signal changes dominate at ultra-high field (UHF) strengths (Boxerman, Hamberg, et al., 1995;Uǧurbil et al., 1999;Uludaǧ et al., 2009).

During neuronal activation, excessive oxygenated blood flows to the activated area and through the venous system. The differences between the oxygenated and deoxygenated blood in the veins change T_2_^*^times. This change is usually captured with gradient echo (GRE) sequences. In addition, higher blood flow and blood volume in an active brain area increase T_2_times due to higher diffusion of spins in this area. Diffusion effects are more prominent around small vessels, because the diffusion distance relative to the vessel size is higher (Weisskoff et al., 1994). Spin echo (SE) sequences highlight this effect. They are not sensitive to spin dephasing around veins with a diameter way larger than the diffusion length, due to their refocusing pulse. With increasing field strength, the T_2_effect is increased relative to the T_2_^*^contrast (Yacoub et al., 2001). Thus, it can be concluded that SE sequences are more sensitive to smaller veins and, therefore, more spatially specific, particularly at high and UHF strengths.

Echo-planar imaging (EPI) is the earliest sequence used for high-speed image acquisition (Mansfield, 1977;Stehling et al., 1993). It also has a high BOLD sensitivity, making it the most widely used readout in fMRI (Belliveau et al., 1991;Ogawa et al., 1990). However, it suffers from geometric distortion and susceptibility artifacts increasing with field strength due to B_0_inhomogeneities (Moeller et al., 2010;Speck et al., 2008). Moreover, increased blurring effects are expected with increasing field strength due to faster T_2_^*^decay (Uludaǧ et al., 2009and references therein). The T_2_^*^weighting of GRE-EPI not only leads to a bias toward large draining veins on the cortical surface (Bause et al., 2020;Lai et al., 1993;Turner, 2002), and a loss of specificity toward the cortical surface in layer fMRI, but also to an increase in sensitivity (Polimeni et al., 2010). SE-EPI, however, is severely limited by the higher specific absorption rate (SAR), which can limit the temporal resolution and/or spatial coverage especially at field strengths≥7T (Budde et al., 2014). Therefore, GRE-EPI is still preferred at UHF strengths.

Fortunately, other sequences, like balanced steady-state free precession (bSSFP), may also be utilized to map the BOLD effect at high and UHF strengths (Bowen et al., 2005;Miller, 2012;Scheffler & Ehses, 2016;K. Zhong et al., 2006). This sequence maintains magnetization in the steady state, which makes it highly efficient in this regard. However, due to its lower sensitivity to the BOLD signal, it is not more widely adopted in BOLD fMRI imaging. BSSFP is furthermore slow in comparison with EPI due to the separate excitation of and rewinding in each k-space line. This also results in high gradient duty cycles and a higher SAR than GRE-EPI. In terms of robustness to B_0_inhomogeneities, bSSFP has the benefit, that the images do not suffer from geometric distortions. But in regions where the static field inhomogeneities are too strong, so-called banding artifacts can occur due to incomplete signal refocusing. The strength of this artifact, therefore, also depends on the TR of the sequence. Moreover, the contrast in bSSFP is T_2_/T_1_weighted, and although it is a GRE sequence, echo formation at TE = TR/2 (which is used in this work) is similar to SE, meaning that the transverse magnetization is (nearly) refocused when the echo is acquired (Scheffler & Hennig, 2003). In theory, the SE-like signal of bSSFP shows increased selectivity to the microvasculature at high fields (Bowen et al., 2005) with a high intravascular contribution (Pérez-Rodas et al., 2021). This theoretical expectation was further strengthened by Monte Carlo simulations using sections of vascular networks of the mouse parietal cortex (Báez-Yánez et al., 2017). It can, therefore, be expected that bSSFP enables the acquisition of more specific fMRI signals, closer to neuronal activation sites.

With increasing field strength ($B_{0}$), the overall signal-to-noise ratio increases (Pohmann et al., 2016). Smaller voxel sizes are feasible, increasing the heterogeneity of voxels in tissue, vessels, or cerebrospinal fluid (CSF), and reducing physiological signal confounds (Triantafyllou et al., 2005). Moreover, simulations have shown that signal contribution from the microvasculature increases by the square of$B_{0}$(Ogawa et al., 1993), while signal from the macrovasculature increases only linearly. Accordingly, a more specific BOLD effect should occur at UHF compared with lower field strengths. However, as the extravascular effect dominates at UHF, a higher signal contribution from large draining veins can be expected in T_2_^*^-sensitive sequences and thus an increased dependence of signal fluctuation on the cortical orientation relative to$\vec{B_{0}}$. For example, Gagnon et al. showed that the distinct orientation of the cortical macrovasculature is not reflected in the SE signal amplitude (Gagnon et al., 2015), but in the GRE signal by performing simulations of the extravascular signals in vascular models and GRE BOLD measurements during hypercapnia. The concept was then further strengthened byBáez-Yánez et al. (2017)by comparing the cortical and, therefore, vascular orientation dependence of GRE, SE, and bSSFP in simulations. This study showed a BOLD signal change dependence on the cortical orientation that varied with cortical depths for all three sequences and a high signal sensitivity of bSSFP to the microvasculature, similar to SE (Báez-Yánez et al., 2017). The strong orientation dependence of the GRE-EPI at UHF was then demonstrated byViessmann et al. (2019), who investigated the resting-state fMRI signal of 2D simultaneous multi-slice (SMS) GRE-EPI (Setsompop et al., 2012) at 3T and 7T. They observed a much stronger cortical orientation dependence of the fMRI signal fluctuation at 7T than at 3T, calculated as the coefficient of temporal signal variation (CV). Like in previous work (Báez-Yánez et al., 2017;Cohen-Adad et al., 2012;Gagnon et al., 2015;Viessmann et al., 2019),$\theta_{B_{0}}$is defined here as the angle between the cortical surface normal and the main magnetic field$\vec{B_{0}}$(illustrated inFig. 1A). The angular dependence of CV followed a${\text{cos}}^{2}\left(\theta_{B_{0}}\right)$curve, which can be expected for GRE-EPI fromEquation 1at UHF, as$\nu =\frac{\pi }{2}-\theta_{B_{0}}$. For sequences with reduced macrovascular contribution, like bSSFP, the orientation dependence should be lower, since the intravascular effect dominates at 3T and 9.4T, especially in the macrovasculature and for short repetition times (TRs) (Pérez-Rodas et al., 2021).

**Fig. 1.**
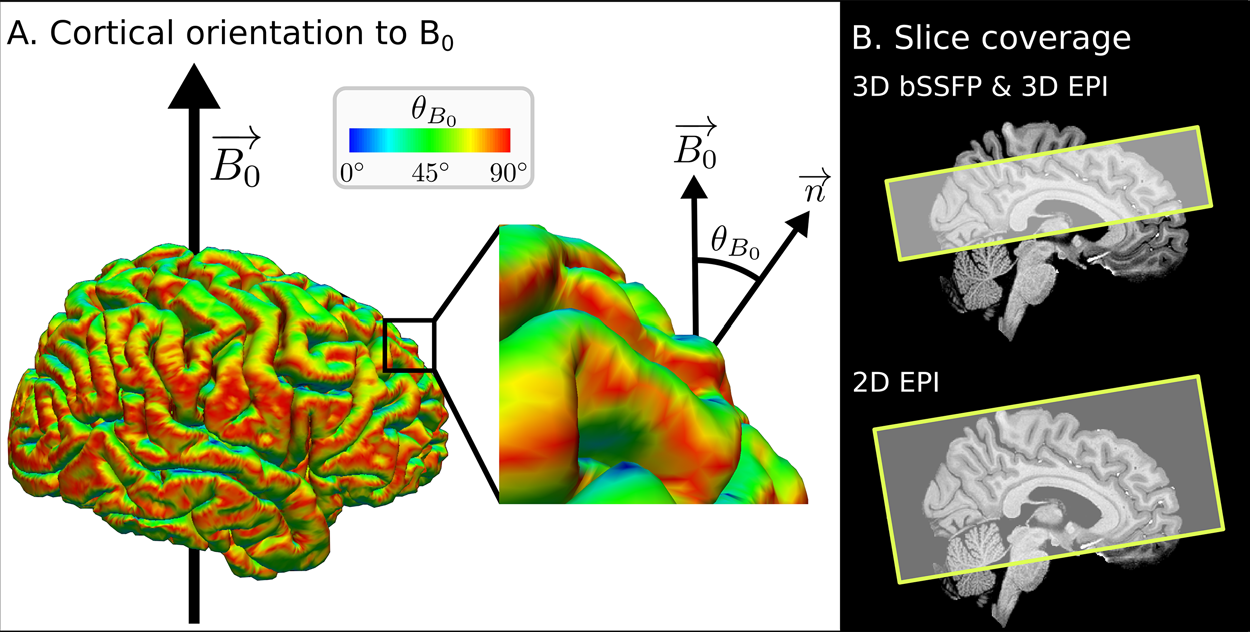
Illustration of$\theta_{{\text{B}}_{0}}$and slice coverage. (A) The cortical orientation to$\vec{B_{0}}$($\theta_{B_{0}}$), which is the angle between$\vec{B_{0}}$and the surface normal, is illustrated on the FreeSurfer result from subject S5. (B) Slabs were acquired parallel to the calcarine sulcus, covering 40 and 44 slices for 3D bSSFP and 3D EPI, respectively. The same orientation and higher slice coverage (87 slices) were chosen for 2D EPI. The exemplary slice coverage is shown on a skull stripped, T_1_-weighted MPRAGE after bias field correction for subject S5. For the analysis, extra slices in 3D EPI and 2D EPI were removed to have the same analyzed region of interest in all three sequences.

To further investigate this phenomenon experimentally, we compare the resting-state fMRI signals of 3D bSSFP (Ehses & Scheffler, 2018) with segmented 3D GRE-EPI (Stirnberg & Stöcker, 2021) and 2D SMS GRE-EPI (vendor provided) in their cortical orientation and cortical depth dependence at 9.4T. In the 2D SMS GRE-EPI examinations, a similar sequence protocol like in a previous study (Viessmann et al., 2019) was applied to allow for better comparison of results at an even higher field strength. Furthermore, we investigated the dependence of the resting-state signal on the cortical orientation in relation to the distance to large veins, expecting a lower orientation dependence when investigating voxels further away from veins.

## 2 Methods

### 2.1 Data acquisition

The presented study is part of a test plan for the development of 9.4 Tesla functional imaging methods reviewed and approved by the Ethics Committee of the University of Tübingen (approval no. 649/2021BO1). The volunteers provided their written informed consent to participate in this study.

MRI data of the brains of six subjects were acquired on a whole-body 9.4T scanner with an in-house built coil (16 Tx/31 Rx) (Shajan et al., 2014). Data from one subject were discarded due to excessive motion. For each subject, two sessions of resting-state fMRI measurements were acquired at the same time of day. In the first session, four runs of each 3D bSSFP (Ehses & Scheffler, 2018) and segmented 3D GRE-EPI (Stirnberg & Stöcker, 2021), both with a volume repetition time (TR_vol_) of 3 s, and one run of 2D SMS GRE-EPI with a TR_vol_of 1.7 s were acquired. The imaging parameters of both 3D sequences were optimized to obtain comparable spatial coverage and TR_vol_. The acquisition time of about 5.5 min was held constant for all sequences by adjusting the number of measured frames. All functional images were acquired with an isotropic voxel resolution of 1.1 mm. The imaging slab of all sequences was oriented parallel to the calcarine sulcus, but almost whole brain coverage was only achieved with the 2D SMS protocol. The orientation and positioning of the slab were chosen to cover as many cortical orientations as possible. The resulting imaging coverage is shown inFigure 1B. In the second session, four runs of 2D SMS GRE-EPI and one run of each 3D bSSFP and segmented 3D GRE-EPI were measured to test for test–retest variability. In both sessions, additional frames of both EPI sequences were acquired with the same parameters and reversed phase encoding (PE) direction to perform unwarping and co-registration of the anatomical data (explained in more detail inSection 2.2.3). Acquisition parameters for all sequences are stated at the end of this section.

Physiological parameters were acquired with a respiration belt and photoplethysmograph for breathing and heart rate tracking, respectively (Biopac Systems Inc., CA, USA). However, physiological noise regression was not performed for all runs due to unstable recordings of the scanner’s trigger signal which hampered the temporal synchronization with the data. Runs with complete physiological recordings were evaluated.

CSF nulled whole-brain T_1_-weighted MPRAGE was acquired during only one session, usually at the end of the first one. For this sequence, a non-selective, population-optimized parallel transmit pulse was used (Bosch & Scheffler, 2023;Gras et al., 2017) in order to improve signal homogeneity. In a third session, 0.48 mm multi-echo (ME) GRE was acquired for the calculation of susceptibility-weighted images (SWI) (Haacke et al., 2010) to estimate the location of large veins. The sequence was modified to allow for the recording of an FID after the acquisition of the last spatially encoded echo for the retrospective correction of physiologically induced phase fluctuations (Vaculčiaková et al., 2022). Unfortunately, one subject (S1) did not meet the requirements to be measured in the third session, which made SWI data acquisition and corresponding analysis for S1 impossible.

The**acquisition parameters**were as follows:

- **3D bSSFP:**nominal matrix size: 174 × 174 × 40, number of volumes: 112, resolution: 1.1 mm isotropic, TR/TE = 3.14 ms/1.57 ms, TR_vol_= 3000 ms, nominal flip angle (FA) = 11°, slice partial Fourier (pF) = 6/8, GRAPPA recon (Griswold et al., 2002), R = 5 × 1, 10% slice oversampling, readout (RO) bandwidth = 1250 Hz/px.
- **segmented 3D GRE-EPI (3D EPI):**nominal matrix size: 174 × 174 × 44, number of volumes: 112, resolution: 1.1 mm isotropic, TR_vol_= 3000 ms, TE = 22 ms, FA = 15°, slice pF = 6/8, GRAPPA recon, R = 3 × 1, PE_1_= anterior-posterior (AP), PE_2_= HF, interleaved multi-shot segmentation factor = 2, nominal echo spacing = 1.07 ms, RO bandwidth = 1106 Hz/px.
- **2D SMS GRE-EPI (2D EPI):**nominal matrix size: 174 × 174 × 87, number of volumes: 197, resolution: 1.1 mm isotropic, TR_vol_= 1700 ms, TE = 25 ms, FA = 60°, acceleration mode = slice acceleration, acceleration factor PE = 4, SMS = 3, PE direction = AP, nominal echo spacing = 0.79 ms, RO bandwidth = 1512 Hz/px.
- **MPRAGE:**nominal matrix size: 256 × 306 × 300, resolution: 0.7 mm isotropic, TR_inversion_= 3400 ms, TR_readout_= 6.5 ms, slice pF = 6/8, GRAPPA recon, R = 2 × 1, FA = 7.5°, TI = 1460 ms, bandwidth = 400 Hz/px.
- **ME-GRE for SWI:**nominal matrix size: 422 × 410 × 176, resolution: 0.48 mm isotropic, TR = 34 ms, TE_1_= 7.58 ms, TE_2_= 15.16 ms, TE_3_= 22.74 ms, TE_4_= 18.2 ms, FA = 16°, GRAPPA recon, R = 2 × 2, RO bandwidth = 213 Hz/px.

### 2.2 Data analysis

#### 2.2.1 Functional data processing

The first two volumes of the 3D EPI and 3D bSSFP measurements were removed to ensure that the analyzed data were acquired in the steady state. As the TR_vol_of 1700 ms for the 2D EPI was shorter than for the 3D sequences (3000 ms), three instead of two volumes were discarded. This resulted in 110 and 194 volumes for further processing of the 3D sequences and the 2D EPI, respectively.

Motion correction was performed in SPM12 (v7771) registering all volumes of all runs of one session to the mean volume of that session to minimize interpolation bias. Second degree B-spline interpolation and no up-sampling were used to perform this step. The full width at half maximum (FWHM) of the Gaussian smoothing kernel applied to the data to estimate realignment parameters was set to 1 mm.

The CV was calculated voxel wise from the motion-corrected volumes as a measure of TR-normalized time series signal fluctuation:

$$CV=\frac{1}{tSNR_{eff}}=\frac{\sigma }{\mu }\sqrt{{\text{TR}}_{vol}}.$$ (3)

Here, tSNR_eff_is the tSNR efficiency, given by the time series mean,μ, divided by the time series standard deviation,σ, normalized by the square root of the volume TR. This normalization was performed in order to better compare CV of the 3D sequences (TR_vol_= 3 s) with the 2D EPI (TR_vol_= 1.7 s).

Distortion correction was performed only on images acquired with the EPI sequences using FSL’s TopUp (6.0.5.2) (Andersson et al., 2003;Smith et al., 2004). The inversion of the resulting field map was used to warp the anatomical data and the values were calculated in the anatomical space, namely (a) the orientation values in voxel space, (b) the ribbon file (gray matter (GM)–white matter (WM) segmentation), and (c) the vein distance maps to the respective EPI space (explained in more detail inSection 2.2.3). It is important to note that TopUp was only used to unwarp the EPI image temporarily and thus be able to co-register the anatomical image to the distortion-corrected EPI and to obtain the field maps to finally warp the anatomical images to the distorted functional space. All further analysis steps were performed in the functional space.

#### 2.2.2 Anatomical data processing

The T_1_-weighted MPRAGE was first bias field corrected in SPM12 using the segment tool, 40 mm FWHM and medium regularization. The bias field-corrected images were then skull stripped to get a brain mask using the integrated synthstrip method in FreeSurfer mri_synthstrip (Hoopes et al., 2022), as this skull-stripping method resulted in a better GM-WM segmentation than the regular one used in the recon-all pipeline of FreeSurfer (v.7.4.0). The latter pipeline was then used to get a segmented brain and mainly two surfaces, the white surface (between WM and GM) and the pial surface (between GM and CSF). As surfaces consist of vertices, and three vertices form a closed surface, the surface normal from each small surface was calculated as explained inViessmann et al. (2019). These$\theta_{B_{0}}$values, which are calculated on the surface (Fig. 1A), were then transformed into voxel space at the native resolution of the MPRAGE of 0.7 mm.

#### 2.2.3 Co-registration and depth segmentation

The T_1_-weighted volume, the cortical ribbon, and orientation values calculated in FreeSurfer were co-registered to the functional volumes as follows: First, the T_1_-weighted MPRAGE was linearly co-registered to either the mean bSSFP volume or the distortion-corrected mean EPI volume in SPM12 by maximizing the entropy correlation coefficient. Second, the cortical ribbon and the orientation values were transformed accordingly using the resulting transformation matrix. Third, an additional warping step using the calculated field map from TopUp was performed on all such co-registered anatomical data to match the distortions in the respective EPI data. The last step was skipped when matching the anatomical data to the bSSFP data, as the line-per-line acquisition does not result in susceptibility-induced distortions along the phase encode direction. After co-registration (and warping), the data were up-sampled by a factor of 4 in all three dimensions using nearest neighbor interpolation. Finally, five equi-distant cortical depths were calculated in LayNii (v.2.6.0) (Huber et al., 2021) from the co-registered (and warped) cortical ribbon. Why up-sampling is necessary before calculating the depths in LayNii is shown inSupplemental Figure S1.

#### 2.2.4 SWI and vein mask

The ME-GRE data were reconstructed offline using in-house developed Matlab (The MathWorks Inc., Natick, MA, USA) routines. The temporal variations of the phase measured by the FID were first subtracted from the corresponding k-space lines using the median FID phase over time as a reference. After GRAPPA reconstruction, complex image data were obtained using the adaptive combine approach (Walsh et al., 2000). Afterward, brain extraction was performed from the resulting images using FreeSurfer mri_synthstrip before SWIs were calculated by repetitive application of a sigmoid filter of the phase data (Santiesteban et al., 2006). The SWIs of the different echoes were then combined as a sum-of-squares to improve contrast, especially for small veins. The resulting “vessel-weighted” image was used as an input for a Matlab implementation of the Jerman vessel filter (Jerman et al., 2016). The resulting data were then thresholded before the vessel mask underwent careful manual inspection in FreeView using the second echo of the ME-GRE as a reference. Particularly at the cortical surface and between the cerebral hemispheres, it was necessary to remove falsely detected vessels. Note that by combining the different echo times, the resulting vascular mask was very conservative since veins appear larger in later echoes due to increased intravoxel dephasing.

Similarly as for the T_1_-weighted data, the vein mask and the ME-GRE were co-registered to the functional data: First, the second echo was co-registered to either the distortion-corrected EPI volumes or the mean bSSFP image. The obtained transformation matrix was then used to re-sample the vein distance map to the (distortion-corrected) functional space. Lastly, these vein distance maps were warped in order to match the respective EPI distortions in the PE direction. Since the vein distance maps were provided in a continuous scale, the data were binned to include all voxels within high (0–0.71 mm), medium (0.71–1.41 mm), and low (1.41–2.12 mm) proximity to the veins. Voxels with a value of 0 mm are located inside the veins.

## 3 Results

### 3.1 Data quality and segmentation

**Fig. 2.**
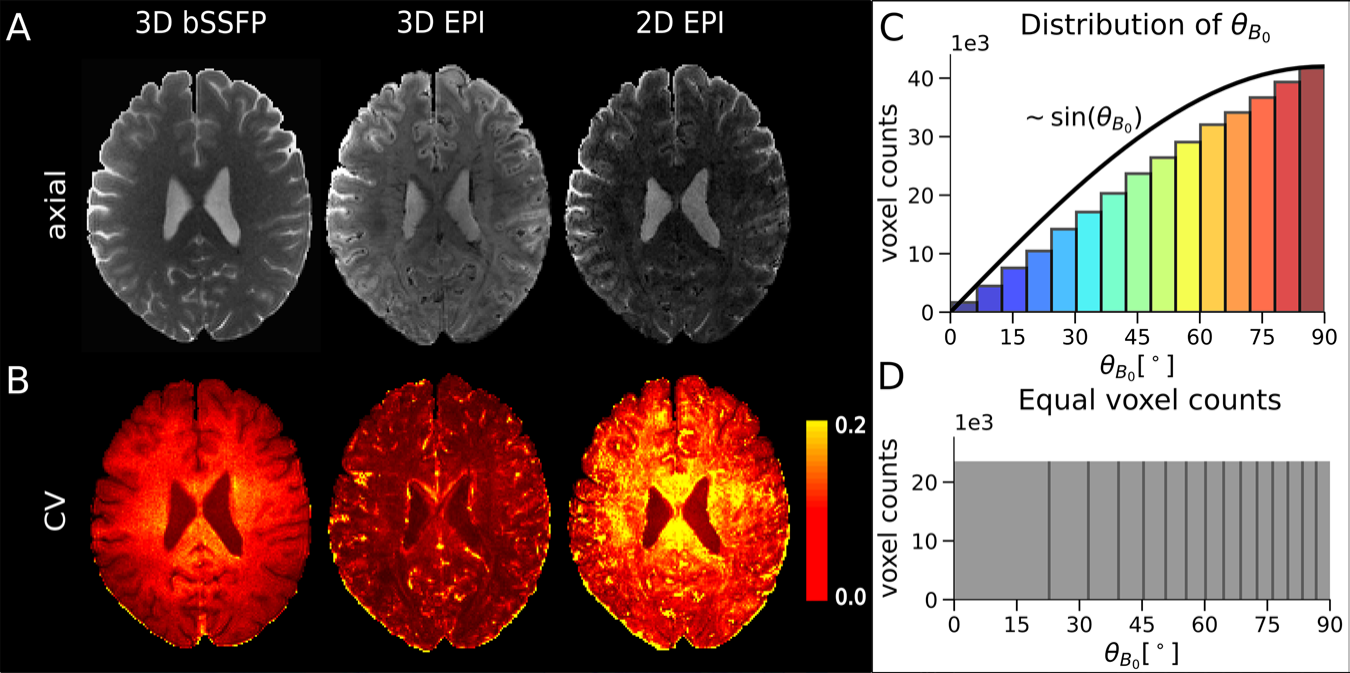
Exemplary axial slices, CV, and distribution of$\theta_{B_{0}}$.*(A) Exemplary*slices from one run of subject S5 for 3D bSSFP (left), segmented 3D EPI (middle), and 2D SMS EPI (right) before motion correction. (B) The CV values calculated from the time series of the respective sequences after motion correction. (C) Distribution of$\theta_{B_{0}}$in the entire cortical ribbon after co-registration to the bSSFP sequence. It can be seen that the distribution follows a sine curve. In order to perform the analysis without a higher weighting of voxels around 90°, the orientation intervals are adjusted as shown in (D) for further analysis. The distributions of$\theta_{B_{0}}$after co-registration and warping to the EPI sequences, and the respective images are shown inSupplemental Figure S2.

Data quality is shown inFigure 2Afor each sequence. An exemplary axial slice of one run from subject S5 and corresponding CV values are presented (Fig. 2B). It can be seen that 3D EPI shows lowest CV values (highest tSNR efficiency), followed by 3D bSSFP and 2D EPI. For bSSFP, CV values in GM are lower than in WM, reflecting higher tSNR values in GM. All three sequences have CV values in similar ranges, which makes a comparison between the different sequences feasible. The distribution of the cortical orientation values to$\vec{B_{0}}$after co-registration (and warping) is shown inFigure 2C. It can be seen directly that more voxels have a$\theta_{B_{0}}$value around 90°, which are located in a cortical region with an orientation parallel to the main magnetic field. This is expected and can be explained by the distribution of angles for randomly located points on the surface of a sphere, which can be described by a sine curve. Due to the unequal number of samples for the different angles, however, this distribution would lead to a strong weighting of higher$\theta_{B_{0}}$values, which in turn would bias the data analysis. To remove this bias, the cortical orientation bin sizes are adjusted accordingly, so that each point in each plot of the following figures corresponds to a similar number of voxels. An example of how bins are adjusted is shown inFigure 2D.

### 3.2 Cortical orientation and cortical depth dependence

The cortical depths,$\theta_{B_{0}}$, and the CV values in the functional space were used to compare the signal fluctuation of all three sequences with respect to their dependence on both the cortical orientation to$\vec{B_{0}}$and the cortical depths. In order to have matching spatial coverage of 36 slices for all 3 sequences, the outer most slices were removed symmetrically (2, 4, and 25 from 3D bSSFP, 3D EPI, and 2D EPI, respectively). For each cortical region of interest, the mean of the CV values of all runs within each (adjusted) cortical orientation bin,$\bar{CV}\left(\theta_{B_{0}}\right)$, is normalized to the corresponding mean within the$\theta_{B_{0}}∼90^{\circ }$bin, yielding the relative CV:

$$CV_{rel}=\frac{\bar{CV}\left(\theta_{B_{0}}\right)-\bar{CV}\left(\theta_{B_{0}}∼90^{\circ }\right)}{\bar{CV}\left(\theta_{B_{0}}∼90^{\circ }\right)}.$$ (4)

This normalization is performed as the lowest signal fluctuation is theoretically expected and found in voxels with$\theta_{B_{0}}∼90^{\circ }$(vessels parallel to$\vec{B_{0}}$).

Voxels from similar slice positions from all subjects were investigated and results from all sequences in session 1 are shown inFigure 3, which displays the relative CV versus cortical orientation in the entire cortical ribbon (first row) and in each of the five cortical depths (rows 2–6). Different markers denote different subjects and the mean of all five subjects is shown in blue. Due to the adjusted$\theta_{B_{0}}$bin size per subject, each plotted point includes equal voxel numbers, as shown inFigure 2D. The mean$\theta_{B_{0}}$over all sequences plotted in the cortical ribbon to get equal voxel counts per bin was as follows: 14.04, 26.57, 34.41, 40.90, 46.68, 52.01, 57.00, 61.68, 66.09, 70.29, 74.27, 78.04, 81.61, 85.04, and 88.36. Plotted CV values for the different sequences in the different cortical depths are given inSupplemental Table S1.

**Fig. 3.**
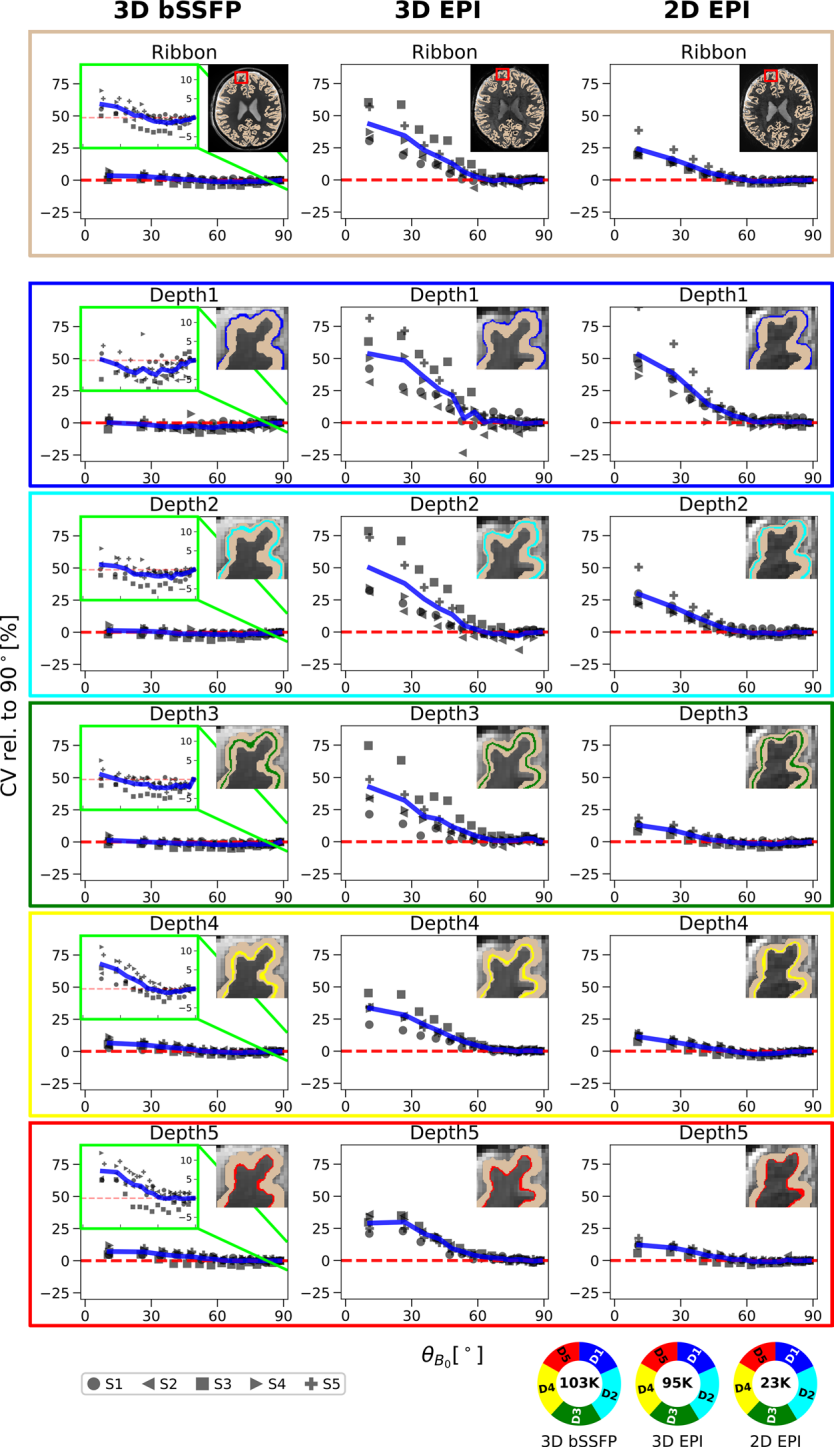
Relative coefficient of variation in the up-sampled data with respect to the cortical orientation to$\vec{{\text{B}}_{0}}$for 3D bSSFP (left), 3D EPI (middle), and 2D EPI (right): The mean CV_rel_values calculated from all runs of each subject in session 1 are plotted against the mean$\theta_{B_{0}}$of each range in the entire cortical ribbon (top row) and in each cortical depth (rows 2–6). Depth 1 and Depth 5 are closest to CSF and WM, respectively, as shown in the upper right corner of each plot. The CV values are plotted relative to the value at$\theta_{B_{0}}=90^{\circ }$(CV_rel_) indicated by the red dashed line at 0%. The blue line shows the mean CV_rel_across all subjects. Due to the lower variability of the CV in bSSFP, zoomed-in details are shown in the green boxes for a decreased range of −8% to 14%. The pie chart at the bottom shows the average number of data samples per point in the cortical ribbon plot across subjects. The distribution of voxels in each cortical depth is represented by the colors.

For both EPI sequences, a strong dependence of the relative CV values on$\theta_{B_{0}}$is visible. Furthermore, the largest relative CV values and the strongest inter-subject variability especially in the shallow cortical depths can be seen for 3D EPI. In the bSSFP data, no such dependence is observed and the CV values are barely affected by the cortical orientation at any depth. The inset plot for bSSFP (green frame) shows the relative CV values on a smaller scale (in the range of −8% to 14%) across the entire orientation range to appreciate the small orientation dependence that is yet present.

In Depth 1 (closest to the cortical surface), the mean CV value over all subjects at approximately$\theta_{B_{0}}=14^{\circ }$is 0.19%, 53.59%, and 53.06% greater than the value at approximately$\theta_{B_{0}}=90^{\circ }$for 3D bSSFP, 3D EPI, and 2D EPI, respectively. At Depth 5 (closest to WM), this value increases slightly to 7.16% for 3D bSSFP, whereas a decrease in orientation dependence can be seen for 3D EPI (29.16%) and 2D EPI (12.12%). Thus, the largest decrease in orientation dependence of CV for increasing cortical depths is noticeable in the 2D EPI plots (decrease of almost 41%). The mean and standard deviation of the time series of all sequences plotted against$\theta_{B_{0}}$are shown inSupplemental Figure S3.

The pie chart inFigure 3shows the mean number of data samples across subjects for each plotted point in the entire cortical ribbon. For the analysis, only the data acquired during the first session are considered, which explains the lower number of voxels for each plotted point in the cortical ribbon for 2D EPI (one run) compared with the 3D sequences (four runs). The inverse number of runs was acquired in session 2, where a comparable behavior for the different cortical orientation dependence of CV is shown (Supplemental Figure S4).

To further investigate the depth dependence observed inFigure 3, the mean of all CV values of each session is plotted against the cortical depths without normalization to$\bar{CV}\left(\theta_{B_{0}}∼90^{\circ }\right)$inFigure 4A. Here, absolute values are shown in the top row, while values relative to D5 are shown in the second row. D5, which is closest to WM (and thus furthest from large draining veins), is used for data normalization, as it shows the least variance across orientations compared with the other depths. Markers are color coded depending on their cortical orientation, with lighter orange indicating higher$\theta_{B_{0}}$values. A tendency of$\theta_{B_{0}}∼\text{90°}$to have a lower dependence on the cortical depth is shown in both EPI plots. The highest dependence of the signal fluctuation on the cortical depth is shown in the 3D EPI plot. For the 2D EPI, the dependence is much lower but a strong variability across cortical orientations is still visible. As already visible inFigure 3, the bSSFP data show much lower CVs and a reversed trend in depth dependence. In addition, the influence of the cortical orientation on CV appears much lower for bSSFP.

**Fig. 4.**
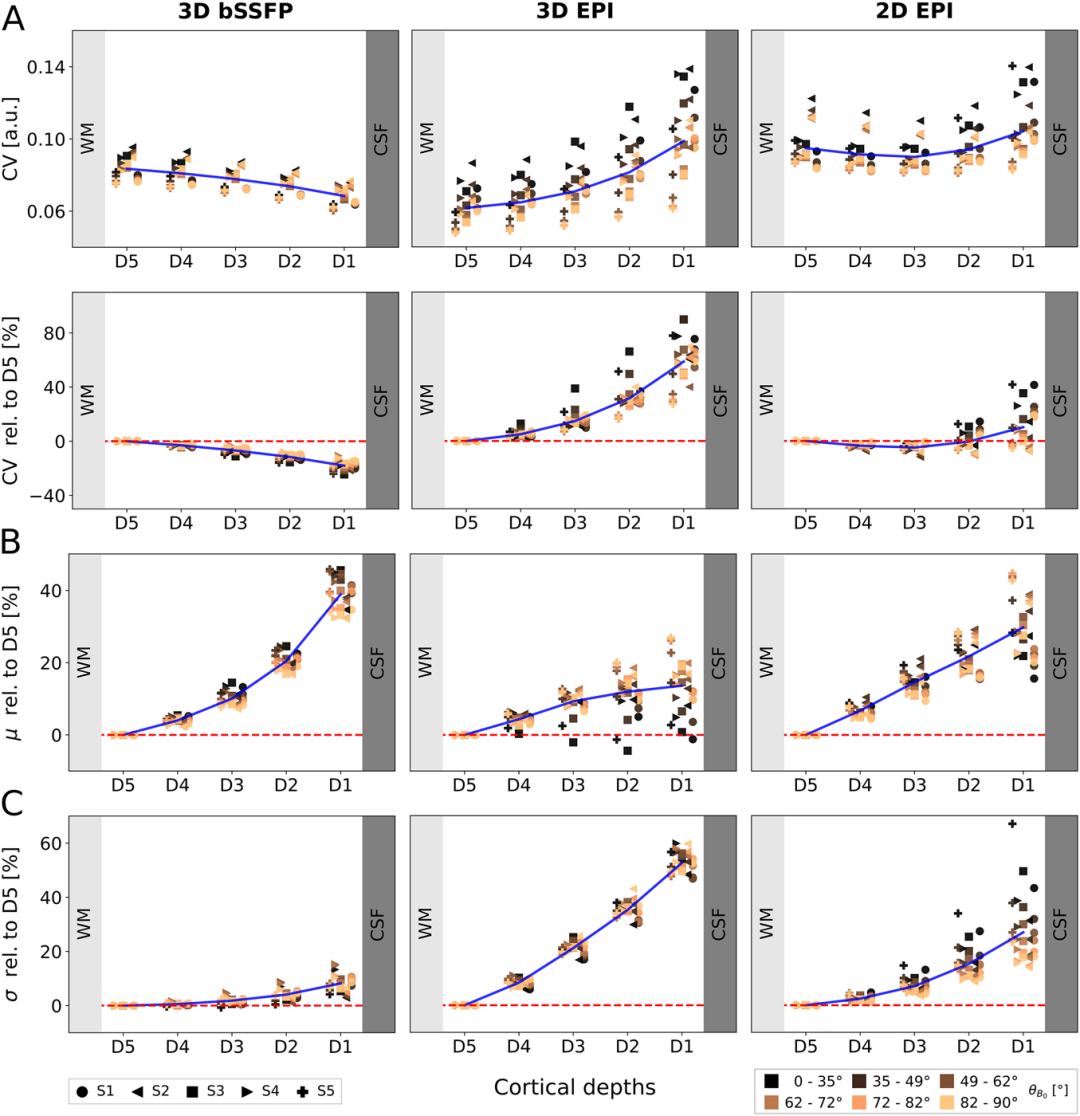
CV,μ, andσplotted against the cortical depth for 3D bSSFP (left), 3D EPI (middle), and 2D EPI (right). (A) The absolute CV values (top) and relative to D5 (bottom) are plotted against the cortical depth with D1 and D5 closest to CSF and WM, respectively. Each point represents the mean of all CV values within a specific range of$\theta_{B_{0}}$values in one session. Six ranges are plotted for each subject, with lighter orange corresponding to higher$\theta_{B_{0}}$values around 90°, see legend. The intervals are not constant, so that each plotted point includes the same number of voxels for each subject. The mean of all subjects is shown in blue. The mean (B) and the standard deviation (C) of the time series are plotted similarly against the cortical depth.

The mean (signal amplitude) and standard deviation (signal variance) of the time series, which both contribute to CV, are plotted against the cortical depth (Fig. 4B and C) relative to D5. For 3D bSSFP, the mean increases almost exponentially toward CSF and the standard deviation increases only slightly. For 3D EPI, the mean increases toward CSF in an orientation-dependent manner, with$\theta_{B_{0}}∼\text{0°}$showing almost no increase. For one subject (S3), these values even decrease and then increase toward CSF. The average standard deviation is 52.6% higher in D1 than in D5. For 2D EPI, the mean signal increases almost linearly toward CSF without any dependence on the cortical orientation, while the standard deviation also increases toward CSF, but with a higher slope for$\theta_{B_{0}}=\text{0°}$than for$\theta_{B_{0}}=\text{90°}$and a stronger inter-subject variability.

### 3.3 Influence of proximity to large veins

**Fig. 5.**
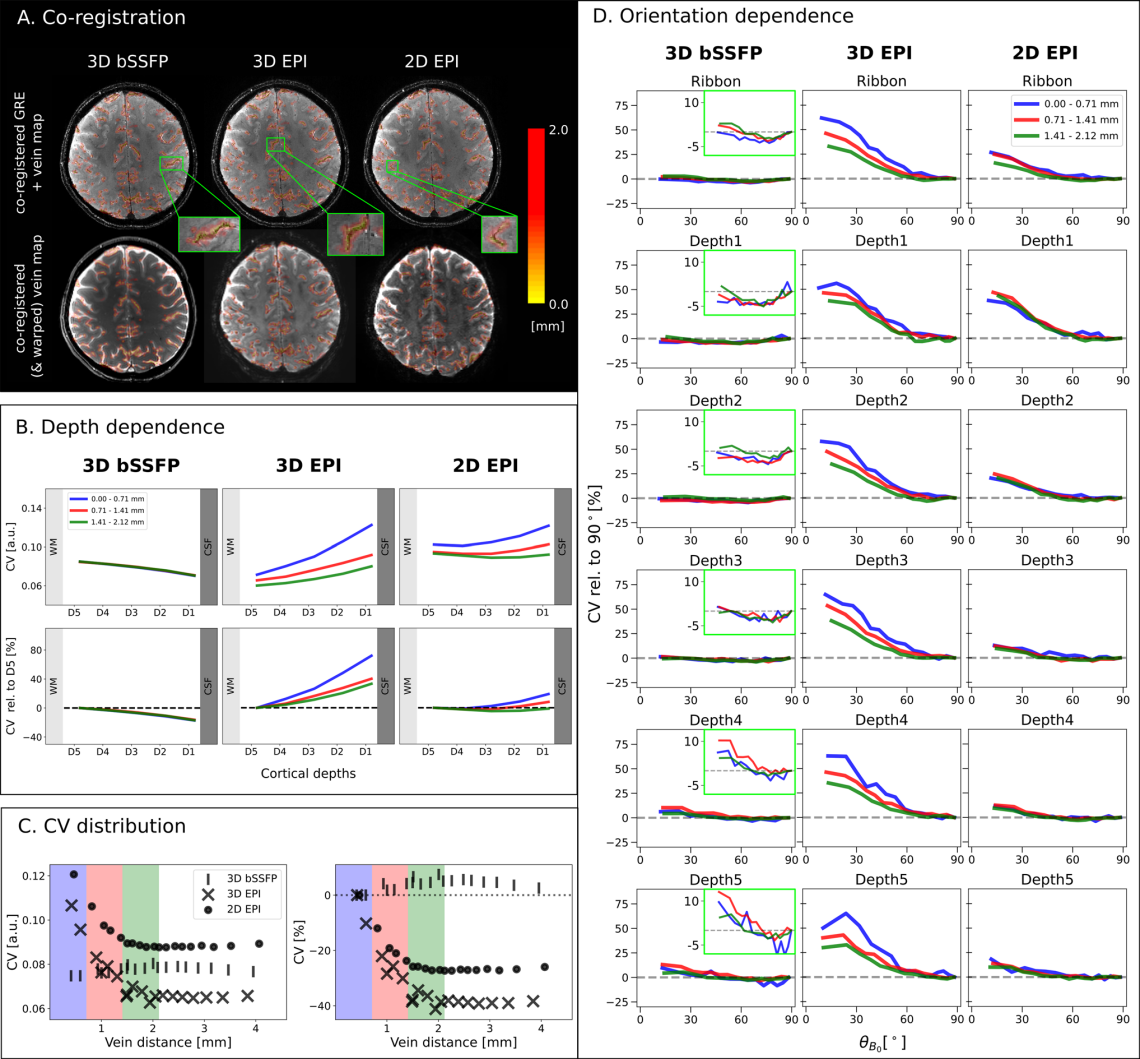
Effect of vein distance. (A) Vein distance map shown on the co-registered gradient echo volume from which they were calculated (top), and overlaid on the mean functional images after co-registration (and warping) (bottom). (B) Average depth dependence of CV (seeFig. 4Afor details) relative to three distances to veins. Vein distances of 0–0.71 mm, 0.71–1.41 mm, and 1.41–2.12 mm are represented by the blue, red, and green lines, respectively. The mean CV of four subjects is plotted against the cortical depth. (C) CV plotted against vein distance for all sequences. The absolute values (left) and relative to the voxels at highest proximity to veins (right) are shown. Data points contributing to (B) and (D) are highlighted in similar colors. Within a sequence, every marker represents the same number of voxels (340 K, 320 K, and 300 K for 3D bSSFP, 3D EPI, and 2D EPI, respectively). (D) Average cortical orientation dependence (seeFig. 3for details) in three distances to veins. Color coding similar to (B).

According to the shown results, it should be clear that removing voxels in proximity to large draining veins will decrease the effect of cortical orientation dependence if a sequence is highly sensitive to these large veins.Figure 5Ashows the co-registered results of the vein distance maps on the second echo of the ME-GRE volume and on the temporal mean volume of the functional volumes. We show the cortical orientation dependence of three pools of voxels inFigure 5Dfor subjects S2–S5 since the venous vessel mask was only available from these subjects. The blue line includes voxels within detected veins and with distances up to 0.71 mm (high proximity) from the veins. The green and the red lines include voxels, at 0.71–1.41 mm (medium proximity) and 1.41–2.12 mm (low proximity) distance from veins, respectively.

The effect of vein distance on the cortical orientation dependence is particularly remarkable in 3D EPI. When looking at the pool of voxels further away from the veins, the cortical orientation dependence decreases. This can not only be seen in the entire cortical ribbon, but also in each cortical depth. In the cortical ribbon, the CV value at approximately$\theta_{B_{0}}=14^{\circ }$is 61.89% higher than the value at$\theta_{B_{0}}=90^{\circ }$for voxels in high proximity to veins. This value is reduced by 15.67% and 28.98% for voxels in medium (red) and far distance (green) to the veins, respectively. Only when looking closely, a decrease of orientation dependence is also visible in 2D EPI, and this only in the entire cortical ribbon (when averaging all depths). Here, the value at$\theta_{B_{0}}=14^{\circ }$closest to veins is 26.74% higher than the$\theta_{B_{0}}=90^{\circ }$. This value is decreased by only 1.95% and 10.85% for voxels at medium and low proximity, respectively. In bSSFP, this effect is slightly reversed, with the lowest value shown for voxels closest to veins. The value at approximately$\theta_{B_{0}}=14^{\circ }$is 0.15% lower than the value at$\theta_{B_{0}}=90^{\circ }$for voxels closest to veins. This is increased by 2.34% and 2.99% for voxels in medium and low proximity, respectively.

In addition, absolute CV values and CV values normalized to D5 from voxels binned in the same manner as explained above were plotted with respect to the cortical depth (Fig. 5B). As expected, the CV values exhibit a greater increase toward the cortical surface when voxels are in proximity to veins for both EPI sequences. In 3D EPI, the highest signal fluctuation is seen for voxels of D1 when closest to veins. This effect is reduced, but is still prominent for voxels with distances 1.41–2.12 mm from the veins. In 2D EPI, the green curve (furthest from veins) even almost becomes flat. This clearly demonstrates that the high signal fluctuation in the EPI sequences on the cortical surface is partly of venous origin. Still the slope in 3D EPI is significantly higher than that in 2D EPI when moving away from the veins. In contrast, the 3D bSSFP plot across cortical depths does not change for any of the voxel pools. Whether voxels are close to a vein or further away, the pattern of CV dependence is almost identical and varies little across cortical depths, with the lowest CV found in voxels located on the cortical surface.

InFigure 5Cwe show the CV of all three sequences plotted as a function of the vein distance (absolute and relative to the value at smallest vein distance). It is expected that voxels closer to the veins fluctuate more, thus have a higher CV value for the T_2_^*^-weighted sequences. For 2D and 3D EPI, the absolute CV values are highest when closest to large veins, which is not the case for 3D bSSFP. This once again highlights the sensitivity of the GRE-EPI to large veins, which is not seen for bSSFP.

### 3.4 Inter-session stability

**Fig. 6.**
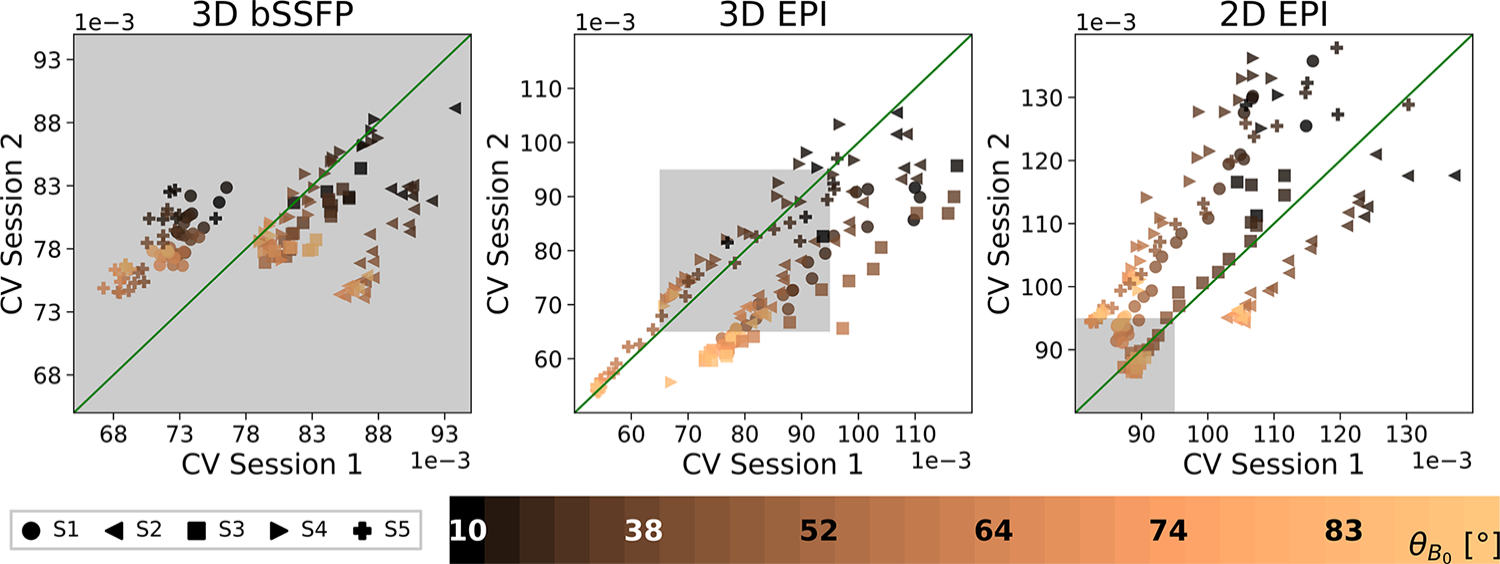
Inter-session variability*.*Mean CV values from run 1 of session 2 are plotted against mean CV values from run 1 of session 1 for all subjects. Thirty$\theta_{B_{0}}$ranges are shown for each subject with their mean$\theta_{B_{0}}$value shown in the color bar for every fifth range. The green line represents the ideal case, where both sessions are indistinguishable. Correlation coefficients are listed inTable 1. Note that each of the sequence plots has different x and y ranges. The range shown in the bSSFP plot is highlighted in the EPI plots as a gray box.

Until here, we only show the cortical orientation (Figs. 3and5D) and cortical depth (Figs. 4and5B) dependence of the signal fluctuations in one session. The results from the second session are shown inSupplemental Figures S4 and S5(with vein distance inSupplemental Figures S6 and S7). To investigate the repeatability of our observations, we plot the mean CV values of one run from session 2 against the mean CV values of one run from session 1 for all subjects (Fig. 6). To this end, the coefficient of variation for 30 ranges of$\theta_{B_{0}}$(indicated by color) were extracted from 1 run of each session and plotted against each other. Each cortical angle range has the same number of voxels. The EPI plots show a high repeatability between the different sessions. This is also expressed in the correlation coefficients for individual subjects (Table 1), where a high repeatability between the sessions is shown, especially for 2D and 3D EPI. The bSSFP plots show a lower repeatability, which might be explained by the small range of CV values (shown as a gray box in all plots). Thus, the correlation coefficients of the CV as function of$\theta_{B_{0}}$for bSFFP only range between 0.77 and 0.96 compared with 0.94–0.98 for the two EPI sequences. Nevertheless, the obtained correlation coefficient values indicate a high repeatability of the cortical angle dependence of the CV within subjects and sequences.

**Table 1.** *Correlation coefficient*of the CV values plotted inFigure 6calculated for each subject and sequence between sessions 1 and 2 using the first run of each.

	**S1**	**S2**	**S3**	**S4**	**S5**
**3D bSSFP**	0.77	0.93	0.87	0.96	0.84
**3D EPI**	0.97	0.96	0.94	0.95	0.99
**2D EPI**	0.98	0.97	0.98	0.94	0.96

## 4 Discussion

In this study, coefficient of variation values of resting-state fMRI signals from three sequences acquired across multiple runs and subjects were compared at 9.4T. Specifically, the dependence on (a) the cortical orientation relative to$\vec{B_{0}}$, (b) the cortical depth, and (c) their distance to veins was investigated. To this end, we first examined these influences on 2D SMS EPI at 9.4T with the same parameters used byViessmann et al. (2019)at 7T to compare our results with the existing literature. Imaging parameters of the 3D EPI and 3D bSSFP sequences such as coverage, temporal, and spatial resolution, as well as excitation pulse duration and bandwidth-time-product were set to nearly identical values in order to minimize potential bias. Although results from both EPI sequences were not congruent, a strong dependence of their signal fluctuations on cortical orientation was observed, allowing for a relative comparison between EPI and bSSFP signal fluctuations. According to our results, the cortical orientation to$\vec{B_{0}}$has almost no effect on the signal fluctuations from the bSSFP sequence. The observed CV variation is at least one order of magnitude smaller and follows different curves as a function of cortical orientation, cortical depth, and vein distance compared with the EPI sequences. This may be an indicator of a higher spatial specificity of the bSSFP signal, showing that the signal is sensitive to the microvasculature and not to large pial veins on the cortical surface responsible for this effect. The following sections discuss factors that might affect the results and comparison with existing literature.

### 4.1 Methodological challenges and considerations

Here, we investigate the rs-fMRI signal of three sequences. The global brain activation in rs-fMRI is advantageous for this work’s objective, as it allows larger parts of the brain to be studied simultaneously compared with typical task-based fMRI paradigms, limited only by sequence and acquisition parameters. Another way to obtain large-scale changes of blood oxygenation signal changes could be through hypercapnic tasks such as breath-holding or even light breathing challenges using moderate levels of O_2_reduction/CO_2_increase (Bright et al., 2009;Kastrup et al., 1998;Pinto et al., 2021). However, due to subject-specific breathing patterns, as well as increased subject movement during deep inhalation and exhalation required for the breath-hold task, such experiments are usually difficult to perform and evaluate. Furthermore, hypercapnia might not only lead to a change of oxygenation in the vascular level, but also to complex physiological changes such as decreased metabolism and neuronal activity which makes the interpretation of signal changes even more complex (Peng et al., 2017). We tried to reduce influences on the rs-fMRI signal by measuring four runs of 5 min each, giving us 20 min per subject for each sequence. But even if we compare two sessions taken at the same time of day, with different number of runs (session 1: 4 × 3D sequences & 1 × 2D sequence; session 2: 1 × 3D sequences & 4 × 2D sequence), we get similar results. Nevertheless, it would be interesting to investigate in the future how the BOLD signal, for example, from a visual task is affected by cortical orientation to$\vec{B_{0}}$using the same sequences used in this study. The challenges would include the smaller brain region being investigated and—in the example of the visual cortex—the limited distribution of cortical orientations in that region.

To make both 3D sequences comparable, the TR_vol_and the voxel size were kept constant, while adjusting the acceleration factors for parallel imaging. Since a higher acceleration factor of R = 5 was used for the 3D bSSFP sequence than for the segmented 3D EPI with R = 3, higher g factor losses can be expected for this sequence which would increase noise variance between voxels in resulting images (Avdievich et al., 2019). However, CV values reflecting the reciprocal of tSNR were comparable between the sequences (seeFig. 2Band absolute CV values inFig. 4A), enabling a valid comparison of their dependence on the cortical orientation and cortical depth. Another point of discussion regarding image acquisition could be the effect of variations in the excitation flip angle due to transmit field inhomogeneities, a common problem in UHF MRI. In this study, all measurements except the MPRAGE scans were performed with a static phase shift between the transmit coil elements corresponding to a circularly polarized mode. Thus, for all rs-fMRI scans, the deviations from the nominal flip angle are comparable within subjects and across sessions. We, therefore, believe that the impact of this effect on the presented results is very limited.

Furthermore, the influence of partial volume effects on our results can only be investigated by changing the voxel size of the functional data in the same session and repeating the analysis. For demonstration purposes, this was performed only for two subjects (S1 and S5) in session 2. We used a voxel size of (0.8 mm)^3^isotropic for both 3D EPI and 3D bSSFP. Imaging parameters were adjusted to achieve this resolution, and two runs of the high-resolution data were acquired. High-resolution data were up-sampled by a factor of 3 to approximately match the up-sampling of the 1.1 mm data. InSupplemental Figure S8, the difference between the 1.1 and 0.8 mm curves is visualized in blue and red, respectively, showing almost no difference between both data sets. The impact of partial volume effects on our results is, therefore, expected to be minimal.

LikeViessmann et al. (2019), we chose the temporal CV as a measure of signal fluctuation. To be able to compare absolute CV values of all three sequences with each other, we additionally normalized it by the$\sqrt{TR_{vol}}$, as the 2D sequence was acquired with a TR of 1.7 s (seeViessmann et al., 2019) and the 3D sequences were acquired with a TR of 3 s. For the major part of our results, however, any global scaling factors were eliminated by using relative CV metrics (with respect to CV at$\theta_{B_{0}}∼90^{\circ }$, with respect to CV at D5, or with respect to CV at the smallest vein distance).

Other factors that may have influenced our results include the segmentation of the brain into GM and WM masks calculated with FreeSurfer, which were then used to divide the cortical band into five equally spaced depths. The co-registration of brain masks and cortical orientation values to the functional space also poses a critical step in the analysis. While the (readout) distortions of MPRAGE and bSSFP were negligible, such that they matched well natively, the (different and non-negligible phase encode) distortions of both EPI data required additional warping of the cortical band before layerification. This step introduced additional interpolation bias compared with the distortion-free bSSFP that cannot be accounted for easily. Therefore, after warping the anatomical tissue masks, the data were visually inspected to ensure a good fit to the functional images.

The cortical orientation values ($\theta_{B_{0}}$) were calculated from the T_1_-weighted MPRAGE, which was acquired in the same session as the shown results for all subjects except subject S1. If$\theta_{B_{0}}$were calculated from a different session, this might add a bias for rotations around the x and y axes. Due to the dimensions of the used coil (Shajan et al., 2014), excessive rotation around these axes was not possible. Nevertheless, the x and y rotations were calculated from the transformation matrices during co-registration. As expected, maximum rotation occurred, when the MPRAGE image was acquired in a different session and was at$\Delta \theta_{B_{0}}=\text{4.7°}$for subject S5 between sessions 1 and 2. Since the visualization was done by averaging over irregular ranges of$\theta_{B_{0}}$to keep the number of data points in each range constant, a small deviation like$\Delta \theta_{B_{0}}=4.7^{\circ }$is hardly noticeable. We furthermore show the results of session 2 of all subjects inSupplemental Figures S4 and S5, which still pointed to the same effects, even when the$\theta_{B_{0}}$values were calculated in a different session. The intersession stability inFigure 6and the correlation coefficients inTable 1were further indicators, that calculating$\theta_{B_{0}}$in different sessions did not affect the outcome.

### 4.2 2D EPI cortical orientation and cortical depth dependence

Viessmann et al. (2019)investigated the signal fluctuation dependence of 2D SMS EPI on the cortical orientation at 7T. An even higher effect is expected at 9.4T, as the susceptibility effect and thus the draining-vein bias of a GRE-EPI sequence become higher with increasing field strengths (Uludaǧ et al., 2009). Although similar parameters were used to acquire the 2D EPI data, a direct comparison with the existing literature is not straightforward. This is due to the fact thatViessmann et al. (2019)used FreeSurfer for depth segmentation from superficial CSF through GM to subcortical WM. Furthermore, the authors performed temporal smoothing, and presented the data without removing the orientation bias from equal orientation bin sizes (Fig. 2C). We used LayNii for depth segmentation of GM only, did not perform temporal smoothing, and present the data excluding the orientation bias (Fig. 2D). Although the inclusion of CSF and WM voxels theoretically allows for the investigation of the CV in regions with different vascular architecture and where no or little functional signal is expected, such additional layers are difficult to define unambiguously due to partial volume effects. Temporal smoothing might be beneficial to reduce thermal noise. However, we abstained from modifying the temporal signal to not introduce any potential interpolation bias to the data. Still, the effect of temporal smoothing and physiological noise reduction was investigated for one subject and is shown inSupplemental Figures S9 and S10. Regressing out physiological noise was not feasible for all runs of all subjects, due to an error during data collection, but has been done for one example subject. Finally, we present our data without the orientation bias, by adjusting the sizes of the orientation bins, facilitating their interpretation without unequal CV precision across cortical orientations.

However, despite the different analysis approach, the cortical orientation dependence of 2D EPI persists at 9.4T and was comparable with the effects observed at 7T. Highest CV values occurred for$\theta_{B_{0}}\to 0^{\circ }$(pial vessels perpendicular to$\vec{B_{0}}$) and decreased toward$\theta_{B_{0}}=90^{\circ }$(Fig. 3). Due to the removal of the orientation bias in our data, fewer data points were plotted around$\theta_{B_{0}}∼0^{\circ }$. Nevertheless, the curves of both 2D and 3D EPI were fitted with$CV=A{\text{cos}}^{2}\left(\theta_{B_{0}}+C\right)$expected fromEquation 1and shown byViessmann et al. (2019). To make sure that our plotting method did not affect the quality of the fit, we performed the fit on eight different curves using the same data from the entire cortical ribbon of all subjects (Supplemental Figure S11). As the quality of the fit did not change dramatically depending on the plotting method (R^2^= 0.91-0.98), we conclude that our curves follow a${\text{cos}}^{2}\left(\theta_{B_{0}}\right)$dependence.

While the orientation dependence was present in all cortical depths, it decreased closer to WM. This is expected for 2D EPI (and 3D EPI), as gradient echo EPI is T_2_^*^weighted and highly biased by the extravascular signals caused by large pial veins on the cortical surface. The CV of 2D EPI decreased slightly and then increased toward CSF (Fig. 4A), as similarly shown byViessmann et al. (2019). It was also shown that CV values from regions with lower$\theta_{B_{0}}$, corresponding to greater perpendicularity of the pial veins, show a greater slope across the cortical ribbon. This is expected when veins are perpendicular to$\vec{B_{0}}$, due to the maximization of their signal contribution. In addition, previous theoretical and experimental studies have shown that large pial veins are responsible for signal bias even at greater cortical depths (Bause et al., 2020;Koopmans et al., 2010;Pais-Roldán et al., 2023;Polimeni et al., 2010;Ress et al., 2007;Siero et al., 2011).

### 4.3 3D EPI versus 2D EPI

Compared with 2D EPI, a higher cortical orientation dependence was observed for 3D EPI signal fluctuation (Fig. 3). Both sequences, however, showed a CV decrease toward WM, reflecting the reduced influence of the large veins near WM compared with the cortical surface. When comparing signal fluctuations from both sequences at different cortical depths (Fig. 4), 3D EPI showed a higher increase toward the cortical surface than 2D EPI, except when the cortex was perpendicular to$\vec{B_{0}}$.

One explanation for the observed discrepancies between 2D and 3D EPI might be that 3D EPI is known to be more susceptible to physiological influences (heart rate and breathing) than (single-shot) 2D EPI (Lutti et al., 2013) due to repeated excitation of the entire slab for Fourier encoding along the slice direction. While 3D bSSFP also employs repeated slab excitations and Fourier slice encoding, 3D EPI combines this with strong T_2_^*^-/susceptibility-weighting (long TE), which leads to image artifacts that show as time series signal fluctuations. This might also explain the high variance in the 3D EPI data (Fig. 4C). After regressing out a major source of physiological image noise for one exemplary subject using RETROICOR (Glover et al., 2000), the orientation dependence is reduced for both 2D and 3D EPI, but the CV dependence remains larger for 3D EPI (Supplemental Figures S9 and S10). Another physiological noise correction approach (RVHR), which is more directly linked to vascular oxygenation changes via respiratory variations (RV) and variations of the heart rate (HR), may be interesting to investigate in this context (Chang et al., 2009). However, it is known that the respiratory and cardiac response functions employed in such corrections vary across brain regions. A careful analysis, which is beyond the scope of the work, would, therefore, be needed to avoid yet another potential cortical orientation bias. Furthermore, we observed stronger signal fluctuations and dynamic geometric distortions in the inferior section (closer to the neck) of the 3D EPI scans which is indicative of its physiological origin. In comparison, movements of tissue borders were not visible in the 2D EPI measurements and the mean of the time series shows an almost linear increase toward the cortical surface (Fig. 4B). This indicates that another BOLD time series correction step not performed on the shown data, that is, regression of motion parameters (and their derivatives) to remove signal variations explainable by motion (Power et al., 2014), may change the overall fluctuation amplitude differently in 2D and 3D EPI. We tested this on one run of all subjects in both sessions and show the results inSupplemental Figure S12, which show a closer similarity in the dependence of both 2D and 3D EPI on the cortical orientation after motion regression. While in 3D EPI there is a decrease of orientation dependence on the cortical orientation in each cortical depth, this is only the case in the superficial depths (D1 and D2) in 2D EPI, bringing the dependencies of both sequences closer to each other.

There is also a difference in the readout bandwidth, coverage, and parallel imaging between the two sequences since the 2D SMS-EPI protocol was designed such that a comparison with the data ofViessmann et al. (2019)can be performed. In contrast, the 3D protocol was optimized with regard to coverage, temporal resolution, and tSNR of the bSSFP sequence. Other parameters that have a potential effect on the CV include TE and echo train duration (τ). In our case, however, we kept both of these parameters almost constant between the EPI sequences. Moreover, images were acquired with different TR_vol_in both sequences (1.7 and 3 s for 2D and 3D EPI, respectively). To explore the impact of this discrepancy, an additional analysis was performed by dropping every second volume in 2D EPI, resulting in an effective TR of 3.4 s. After a comparison between the resulting plots, no difference was seen (results not shown). This rules out the possibility that TR_vol_might be responsible for the +19.54% increase of CV of 3D EPI in the entire cortical ribbon compared with 2D EPI.

In summary, physiological influences or different motion sensitivities might explain the observed differences between the amplitudes of 2D and 3D EPI cortical orientation and depth dependence. Further work is, however, needed to carefully analyze these EPI differences without introducing orientation bias, whereas this work focuses on the differences to the 3D bSSFP sequence.

### 4.4 3D bSSFP cortical orientation and cortical depth dependence

While a clear and high dependence of CV and thus the signal fluctuation of the EPI signals on the cortical orientation relative to$\vec{B_{0}}$was observed, this was not the case for bSSFP, especially in the shallow depths. Surprisingly, the signal fluctuation was similarly dependent on$\theta_{B_{0}}$in the deeper cortical depths (D4 and D5), but with reduced magnitude compared with both EPI sequences (Fig. 3).

Theoretically, the intravascular effect, dominating at UHF in bSSFP—particularly in the macrovasculature—(Pérez-Rodas et al., 2021), will cause a frequency shift which is$\Delta \omega_{intra}∼{\text{cos}}^{2}(\nu )$(seeEquation 2). However, this frequency shift will not result in a dependence of the bSSFP signal on$∼{\text{sin}}^{2}(\theta_{B_{0}})$, as the maximum frequency shift experienced by a change of orientation from a parallel vein to a perpendicular vein is approximately 36 Hz. This frequency shift is negligible compared with the one experienced through a change of T_2_between oxygenated and deoxygenated blood. In the extravascular space, however, T_2_is nearly constant, making a change in orientation more pronounced in the measured signal. Therefore, although the intravascular component contributes largely to the bSSFP signal, it will not lead to its cortical orientation dependence.

The extravascular effect of veins at the GM–WM boundary, which often drain deep cortical layers and run parallel to the cortex before ascending to the cortical surface (Duvernoy et al., 1981), may explain the${\text{cos}}^{2}(\theta_{B_{0}})$dependence seen in Depth 4 and Depth 5. Since the signal dependence on orientation cannot be described by a${\text{sin}}^{2}(\theta_{B_{0}})$at any cortical depth, it can be assumed that the bSSFP measurements were not sensitive to ascending veins, or only to a very limited extent. If both, surface veins and orthogonal intracortical veins, contribute equally to the signal, no dependence on the cortical orientation to$\vec{B_{0}}$is expected. The same is the case if bSSFP rs-fMRI signals are predominantly sourcing from randomly oriented capillaries where, in addition, almost 50% of the signal is intravascular and 50% extravascular (Pérez-Rodas et al., 2021). This would negate any dependence on the vein orientation. We observed this behavior in the zoomed-in version of the bSSFP plots for D2 and D3 (green boxes inFig. 3).

In accordance with the aforementioned observations, the cortical depth dependence plots (Fig. 4) showed a decrease of the coefficient of variation toward the cortical surface for bSSFP in contrast to both EPI sequences. On the contrary,Báez-Yánez et al. (2017)show that the BOLD signal increases toward the cortical surface for all simulated sequences, regardless of whether it is GRE, SE, or bSSFP. Although this is not consistent with our findings regarding the relationship between the coefficient of variation (CV) and cortical depths, we observe the same effects when plotting the standard deviation of the time series against the cortical depths. This means that the course of the CV relative to the cortical depths can only be explained by the much stronger increase of bSSFP signal toward the cortical surface.

Miller et al. (2007)investigated the physiological noise of both transient and passband bSSFP at different TRs and field strengths of both 1.5T and 3T and compared them with matched (in the sense of imaging parameters) GRE sequences. In their work, they reported that passband bSSFP at short TR is less sensitive to physiological noise than GRE. This is also reflected in our data shown by the unchanged cortical orientation and cortical depth dependence curves after physiological noise and motion regression (Supplemental Figures S9, S10 and S12).

The observed cortical orientation dependence in bSSFP indicates that the signal contribution in bSSFP is not dominated by the extravascular component of the macrovasculature. This work would be the first experimental demonstration of the bSSFP’s sensitivity to the microvasculature, which had previously been hypothesized based on Monte Carlo simulations (Báez-Yánez et al., 2017;Pérez-Rodas et al., 2021;Scheffler et al., 2019). A reduced macrovascular signal bias would make bSSFP a good alternative for measuring the BOLD signal at high and UHF, for example, in order to investigate cortical depth-dependent signals in laminar studies.

### 4.5 Comparison with simulation studies

Báez-Yánez et al. (2017)simulated the vessel orientation effect on GRE, SE, and bSSFP at 9.4T. They showed the highest dependence on the vessel orientation for GRE, especially for large draining veins (figure 4 inBáez-Yánez et al., 2017). In the SE simulations, a generally lower dependence was shown, with the highest dependence experienced by the microvasculature (≤10 µm). They furthermore show that this effect in bSSFP was highly dependent on the chosen TR and FA. From the simulated parameters, TR/FA = 5 ms/20° were the closest to our chosen parameters (TR/FA = 3.14 ms/11°). In this case, their bSSFP results showed a similar effect as experienced by the SE sequence; an overall reduced dependence on the cortical orientation. Additionally, signal change from the microvasculature (≈1 to 10 µm) showed a higher dependence on the vessel orientation than vessels with diameters of 50 to 200 µm, which was reversed for the GRE sequence. Unfortunately, the effects of the randomly oriented microvasculature cannot be investigated with our method, as they would cancel each other out. However, we were able to show the lack of orientation dependence in the depths closest to CSF, highlighting the negligible signal contribution from the large draining veins on the cortical surface in bSSFP.

Nevertheless, the above-mentioned signal simulations ofBáez-Yánez et al. (2017)did not exactly match our results (see their figure 8) which may be due to several reasons: First, the BOLD effect was simulated, meaning that two states were compared, while we were looking at the fluctuations of the resting-state signal. Second, the vascular network model used in their simulations was based on vessel segmentation of the mouse parietal cortex. It is, however, known that the ratio of arteries to veins is reversed between mice and humans and that the human cortical vascular network varies much more across brain regions. This is why efforts are being made to generate human vascular artificial networks to simulate the BOLD signal more realistically (Cassot et al., 2006;Hartung et al., 2022;Linninger et al., 2019;Lorthois et al., 2011). Third, in the simulations, deep cortical depths were simulated purely as such, without the effect of partial volumes and potential inflow from WM regions. Fourth, because the WM/GM boundary is not identifiable in the vascular models, it is not clear whether the models used in the simulations cover the entire cortex or only parts of it. Finally, the included pial veins in the mouse vascular model are much smaller with diameters up to 70 µm (according to figure 5 inBáez-Yánez et al., 2017) compared with ranges of 50 to 200 µm in humans (Duvernoy et al., 1983). Thus, in humans they can be even much smaller than the resolution used for the generation of the vessel mask in this work which further complicates the comparison of our calculated vessel distance effects with the simulated ones. For these reasons, it is difficult to compare signal simulation using non-human vascular models with our results.

### 4.6 Influence of large veins

Our results suggest that moving away from large veins detected in the SWI images reduces the orientation dependence experienced by the signal fluctuation in GRE-EPI. If the effects of the large surface veins were completely eliminated, the dependence on the cortical orientation would have to be reversed ($∼{\text{sin}}^{2}(\theta_{B_{0}})$) due to the dominance of signals sourcing from orthogonal ascending veins. However, this effect is not seen in our plots, which means that merely removing voxels inside veins or close to veins will not result in an elimination of their effect on the signal (fluctuation), especially at the used spatial resolution. Previous work also showed that the effects of large veins (≥0.3 mm in diameter) can induce perturbations, which are perceptible even 6.7 mm away from the vein (Huck et al., 2023). Much smaller veins are probably not detectable with our method (Bause et al., 2020;Bollmann et al., 2022). Although the dependence on the cortical orientation relative to$\vec{B_{0}}$was not reversed when moving away from the veins, it was diminished. These findings further enhance the potential of our method to be used for studying the macrovascular contribution to the fMRI signal.

### 4.7 Layer fMRI at UHF

Care should be taken when performing resting-state layer fMRI with GRE-EPI sequences, as it is extremely biased by the large draining veins (Bause et al., 2020;Turner, 2002). This is not only the case at UHF, and efforts exist to remove the large veins contribution, even at 3T (X. Z. Zhong et al., 2024). However, for task-based fMRI, the cortical orientation and cortical depth dependence will not be of extreme importance, as these effects will be minimized due to the comparison of two conditions (task vs. baseline). In this work, we were able to reproduce the observations on the rs-fMRI signal originating from the large draining veins through analysis of the cortical orientation relative to$\vec{B_{0}}$in all cortical depths.

Although our work is performed at 9.4T, it is directly applicable to 7T. Past work has shown lower microvascular contributions to bSSFP at 3T compared with 9.4T through simulations (Pérez-Rodas et al., 2021). In GRE-EPI, however, the cortical orientation effect at 3T has been shown to be weaker than at 7T (Viessmann et al., 2019), because its intravascular contribution is as large or even larger than the extravascular contribution at lower field strengths (Boxerman, Bandettini, et al., 1995;Jochimsen et al., 2004;Song et al., 1996). Our work focuses on the UHF applications of bSSFP, and not on its application in lower fields.Miller et al. (2007)have shown the application of bSSFP at 1.5T and 3T. At lower field strengths, bSSFP requires a longer TE (and TR), as the T_2_times of blood are longer at lower fields. However, the longer TR times will lead to more banding artifacts, as the bandings move closer to each other (distance between them given by 1/TR) (Scheffler et al., 2001). The extravascular contribution to bSSFP increases with decreasing field strength, which may result in a higher dependence on the cortical orientation in deeper layers. However, it is very challenging to perform layer fMRI at field strengths of 1.5T and 3T, due to the decrease in spatial resolution. This is why most layer fMRI studies are performed on 7T scanners (or higher), and among other reasons why more scanners with even higher field strengths are being built for humans (Boulant et al., 2024;He et al., 2020;Ladd et al., 2023).

Vascular space occupancy (VASO) (Lu et al., 2003) has been used as an alternative to the conventional BOLD imaging at UHF (Huber et al., 2014). Although direct measurements of cerebral blood volume change using VASO or cerebral blood flow measurements can provide a much higher signal specificity than GRE-BOLD, they usually suffer from a low contrast-to-noise ratio, increased SAR, and lower temporal resolution limiting their applicability for certain research questions. Based on our results, we believe that the bSSFP sequence may be another good alternative to conventional GRE-EPI sequences, allowing depth-dependent and resting-state fMRI without signal bias due to cortical orientation and geometric distortions. However, future work is needed to assess the signal specificity of bSSFP and EPI sequences using task-based layer fMRI and to improve BOLD signal modeling, for example, by using realistic artificial vascular networks.

## 5 Conclusion

In this study, we have investigated the influence of (a) cortical orientation, (b) cortical depth, and (c) vein distance on resting-state fMRI signals of three different sequences: 3D bSSFP, segmented 3D GRE-EPI, and 2D SMS GRE-EPI. While both GRE-EPI sequences exhibited a high cortical orientation dependence, an elevated signal change toward the cortical surface, and a reduced orientation and depth dependence with increased distance to veins, this was not the case for 3D bSSFP. Thus, all three examined parameters (a–c) indicate reduced macrovascular contributions in rs-fMRI scans with bSSFP compared with GRE-EPI and, therefore, a higher specificity for signal changes originating from small veins within the cortex, closer to neuronal activity. Our work provides the first experimental confirmation of the signal characteristics of bSSFP, previously estimated from Monte Carlo simulations. This makes bSSFP a promising sequence for layer fMRI applications, especially at UHF.

## Supplementary Material

Supplementary Material

## Data Availability

Anonymized and defaced MRI data used in this project will be made available upon reasonable request, subject to the signing of a data sharing agreement.

## References

[b1] Andersson , J. L. , Skare , S. , & Ashburner , J. ( 2003 ). How to correct susceptibility distortions in spin-echo echo-planar images: Application to diffusion tensor imaging . NeuroImage , 20 ( 2 ), 870 – 888 . 10.1016/S1053-8119(03)00336-7 14568458

[b2] Attwell , D. , Buchan , A. M. , Charpak , S. , Lauritzen , M. , MacVicar , B. A. , & Newman , E. A. ( 2010 ). Glial and neuronal control of brain blood flow . Nature , 468 ( 7321 ), 232 – 243 . 10.1038/nature09613 21068832 PMC3206737

[b3] Avdievich , N. I. , Giapitzakis , I.-A. , Bause , J. , Shajan , G. , Scheffler , K. , & Henning , A. ( 2019 ). Double-row 18-loop transceive–32-loop receive tight-fit array provides for whole-brain coverage, high transmit performance, and SNR improvement near the brain center at 9.4T . Magnetic Resonance in Medicine , 81 ( 5 ), 3392 – 3405 . 10.1002/mrm.27602 30506725

[b4] Báez-Yánez , M. G. , Ehses , P. , Mirkes , C. , Tsai , P. S. , Kleinfeld , D. , & Scheffler , K. ( 2017 ). The impact of vessel size, orientation and intravascular contribution on the neurovascular fingerprint of BOLD bSSFP fMRI . NeuroImage , 163 , 13 – 23 . 10.1016/j.neuroimage.2017.09.015 28890417 PMC5857886

[b5] Bause , J. , Polimeni , J. R. , Stelzer , J. , In , M. H. , Ehses , P. , Kraemer-Fernandez , P. , Aghaeifar , A. , Lacosse , E. , Pohmann , R. , & Scheffler , K. ( 2020 ). Impact of prospective motion correction, distortion correction methods and large vein bias on the spatial accuracy of cortical laminar fMRI at 9.4 Tesla . NeuroImage , 208 , 116434 . 10.1016/j.neuroimage.2019.116434 31812715

[b6] Belliveau , J. W. , Kennedy , D. N. , McKinstry , R. C. , Buchbinder , B. R. , Weisskoff , R. M. , Cohen , M. S. , Vevea , J. M. , Brady , T. J. , & Rosen , B. R. ( 1991 ). Functional mapping of the human visual cortex by magnetic resonance imaging . Science , 254 ( 5032 ), 716 – 719 . 10.1126/science.1948051 1948051

[b7] Bollmann , S. , Mattern , H. , Bernier , M. , Robinson , S. D. , Park , D. , Speck , O. , & Polimeni , J. R. ( 2022 ). Imaging of the pial arterial vasculature of the human brain in vivo using high-resolution 7T time-of-flight angiography . eLife , 11 , e71186 . 10.7554/eLife.71186 35486089 PMC9150892

[b8] Bosch , D. , & Scheffler , K. ( 2023 ). FastPtx: A versatile toolbox for rapid, joint design of pTx RF and gradient pulses using PyTorch’s autodifferentiation . Magnetic Resonance Materials in Physics, Biology and Medicine , 37 ( 1 ), 127 – 138 . 10.1007/s10334-023-01134-7 PMC10876762 38064137

[b9] Boulant , N. , Mauconduit , F. , Gras , V. , Amadon , A. , Le Ster , C. , Luong , M. , Massire , A. , Pallier , C. , Sabatier , L. , Bottlaender , M. , Vignaud , A. , & Le Bihan , D . ( 2024 ). In vivo imaging of the human brain with the Iseult 11.7-T MRI scanner . Nature Methods , 21 ( 11 ), 2013 – 2016 . 10.1038/s41592-024-02472-7 39420141 PMC11541209

[b10] Bowen , C. , Menon , R. , & Gati , S. ( 2005 ). High field balanced-SSFP fMRI: A BOLD technique with excellent tissue sensitivity and superior large vessel suppression . ISMRM , 13 , 119 . https://cds.ismrm.org/protected/05MProceedings/PDFfiles/00119.pdf

[b11] Boxerman , J. L. , Bandettini , P. A. , Kwong , K. K. , Baker , J. R. , Davis , T. L. , Rosen , B. R. , & Weisskoff , R. M. ( 1995 ). The intravascular contribution to fMRI signal change: Monte Carlo modeling and diffusion-weighted studies *in vivo* . Magnetic Resonance in Medicine , 34 ( 1 ), 4 – 10 . 10.1002/mrm.1910340103 7674897

[b12] Boxerman , J. L. , Hamberg , L. M. , Rosen , B. R. , & Weisskoff , R. M. ( 1995 ). MR contrast due to intravascular magnetic susceptibility perturbations . Magnetic Resonance in Medicine , 34 ( 4 ), 555 – 566 . 10.1002/mrm.1910340412 8524024

[b13] Bright , M. G. , Bulte , D. P. , Jezzard , P. , & Duyn , J. H. ( 2009 ). Characterization of regional heterogeneity in cerebrovascular reactivity dynamics using novel hypocapnia task and BOLD fMRI . NeuroImage , 48 ( 1 ), 166 – 175 . 10.1016/j.neuroimage.2009.05.026 19450694 PMC2788729

[b14] Budde , J. , Shajan , G. , Zaitsev , M. , Scheffler , K. , & Pohmann , R. ( 2014 ). Functional MRI in human subjects with gradient-echo and spin-echo EPI at 9.4 T: SE-EPI and GRE-EPI at 9.4 T . Magnetic Resonance in Medicine , 71 ( 1 ), 209 – 218 . 10.1002/mrm.24656 23447097

[b15] Cassot , F. , Lauwers , F. , Fouard , C. , Prohaska , S. , & Lauwers-Cances , V. ( 2006 ). A novel three-dimensional computer-assisted method for a quantitative study of microvascular networks of the human cerebral cortex . Microcirculation , 13 ( 1 ), 1 – 18 . 10.1080/10739680500383407 16393942

[b16] Chang , C. , Cunningham , J. P. , & Glover , G. H. ( 2009 ). Influence of heart rate on the BOLD signal: The cardiac response function . NeuroImage , 44 ( 3 ), 857 – 869 . 10.1016/j.neuroimage.2008.09.029 18951982 PMC2677820

[b17] Chu , S. C.-K. , Xu , Y. , Balschi , J. A. , & Springer , C. S. ( 1990 ). Bulk magnetic susceptibility shifts in NMR studies of compartmentalized samples: Use of paramagnetic reagents . Magnetic Resonance in Medicine , 13 ( 2 ), 239 – 262 . 10.1002/mrm.1910130207 2156125

[b18] Cohen-Adad , J. , Polimeni , J. R. , Helmer , K. G. , Benner , T. , McNab , J. A. , Wald , L. L. , Rosen , B. R. , & Mainero , C. ( 2012 ). T2* mapping and B0 orientation-dependence at 7T reveal cyto- and myeloarchitecture organization of the human cortex . NeuroImage , 60 ( 2 ), 1006 – 1014 . 10.1016/j.neuroimage.2012.01.053 22270354 PMC3442114

[b19] Duvernoy , H. , Delon , S. , & Vannson , J. L. ( 1981 ). Cortical blood vessels of the human brain . Brain Research Bulletin , 7 ( 5 ), 519 – 579 . 10.1016/0361-9230(81)90007-1 7317796

[b20] Duvernoy , H. , Delon , S. , & Vannson , J. L. ( 1983 ). The vascularization of the human cerebellar cortex . Brain Research Bulletin , 11 ( 4 ), 419 – 480 . 10.1016/0361-9230(83)90116-8 6652521

[b21] Ehses , P. , & Scheffler , K. ( 2018 ). Multiline balanced SSFP for rapid functional imaging at ultrahigh field . Magnetic Resonance in Medicine , 79 ( 2 ), 994 – 1000 . 10.1002/mrm.26761 28547846

[b22] Gagnon , L. , Sakadžić , S. , Lesage , F. , Musacchia , J. J. , Lefebvre , X. , Fang , Q. , Yücel , M. A. , Evans , K. C. , Mandeville , E. T. , Cohen-Adad , J. , Polimeni , J. R. , Yaseen , M. A. , Lo , E. H. , Greve , D. N. , Buxton , R. B. , Dale , A. M. , Devor , A. , & Boas , D. A. ( 2015 ). Quantifying the microvascular origin of BOLD-fMRI from first principles with two-photon microscopy and an oxygen-sensitive nanoprobe . Journal of Neuroscience , 35 ( 8 ), 3663 – 3675 . 10.1523/JNEUROSCI.3555-14.2015 25716864 PMC4339366

[b23] Glover , G. H. , Li , T.-Q. , & Ress , D. ( 2000 ). Image-based method for retrospective correction of physiological motion effects in fMRI: RETROICOR . Magnetic Resonance in Medicine , 44 ( 1 ), 162 – 167 . 10.1002/1522-2594(200007)44:1<162::aid-mrm23>3.0.co;2-e 10893535

[b24] Gras , V. , Vignaud , A. , Amadon , A. , Le Bihan , D., & Boulant , N. ( 2017 ). Universal pulses: A new concept for calibration-free parallel transmission . Magnetic Resonance in Medicine , 77 ( 2 ), 635 – 643 . 10.1002/mrm.26148 26888654

[b25] Griswold , M. A. , Jakob , P. M. , Heidemann , R. M. , Nittka , M. , Jellus , V. , Wang , J. , Kiefer , B. , & Haase , A. ( 2002 ). Generalized autocalibrating partially parallel acquisitions (GRAPPA) . Magnetic Resonance in Medicine , 47 ( 6 ), 1202 – 1210 . 10.1002/mrm.10171 12111967

[b26] Haacke , E. , Tang , J. , Neelavalli , J. , & Cheng , Y. ( 2010 ). Susceptibility mapping as a means to visualize veins and quantify oxygen saturation . Journal of Magnetic Resonance Imaging , 32 ( 3 ), 663 – 676 . 10.1002/jmri.22276 20815065 PMC2933933

[b27] Hartung , G. , Pfannmoeller , J. , Berman , A. J. L. , & Polimeni , J. ( 2022 ) . Simulated fMRI responses using human vascular anatomical network models with varying architecture and dynamics . https://submissions.mirasmart.com/ISMRM2022/Itinerary/Files/PDFFiles/0682.html

[b28] He , X. , Ertürk , M. A. , Grant , A. , Wu , X. , Lagore , R. L. , DelaBarre , L. , Eryaman , Y. , Adriany , G. , Auerbach , E. J. , Van De Moortele , P.-F. , Uğurbil , K. , & Metzger , G. J. ( 2020 ). First in-vivo human imaging at 10.5t: Imaging the body at 447 MHz . Magnetic Resonance in Medicine , 84 ( 1 ), 289 – 303 . 10.1002/mrm.28131 31846121 PMC7083701

[b29] Hoopes , A. , Mora , J. S. , Dalca , A. V. , Fischl , B. , & Hoffmann , M. ( 2022 ). SynthStrip: Skull-stripping for any brain image . NeuroImage , 260 , 119474 . 10.1016/j.neuroimage.2022.119474 35842095 PMC9465771

[b30] Huber , L. , Ivanov , D. , Krieger , S. N. , Streicher , M. N. , Mildner , T. , Poser , B. A. , Möller , H. E. , & Turner , R. ( 2014 ). Slab-selective, BOLD-corrected VASO at 7 Tesla provides measures of cerebral blood volume reactivity with high signal-to-noise ratio: SS-SI-VASO Measures Changes of CBV in Brain . Magnetic Resonance in Medicine , 72 ( 1 ), 137 – 148 . 10.1002/mrm.24916 23963641

[b31] Huber , L. , Poser , B. A. , Bandettini , P. A. , Arora , K. , Wagstyl , K. , Cho , S. , Goense , J. , Nothnagel , N. , Morgan , A. T. , Van Den Hurk , J. , Müller , A. K. , Reynolds , R. C. , Glen , D. R. , Goebel , R. , & Gulban , O. F. ( 2021 ). LayNii: A software suite for layer-fMRI . NeuroImage , 237 , 118091 . 10.1016/j.neuroimage.2021.118091 33991698 PMC7615890

[b32] Huck , J. , Jäger , A.-T. , Schneider , U. , Grahl , S. , Fan , A. P. , Tardif , C. , Villringer , A. , Bazin , P.-L. , Steele , C. J. , & Gauthier , C. J. ( 2023 ). Modeling venous bias in resting state functional MRI metrics . Human Brain Mapping , 44 ( 14 ), 4938 – 4955 . 10.1002/hbm.26431 37498014 PMC10472917

[b33] Iadecola , C. ( 2004 ). Neurovascular regulation in the normal brain and in Alzheimer’s disease . Nature Reviews Neuroscience , 5 ( 5 ), 347 – 360 . 10.1038/nrn1387 15100718

[b34] Iadecola , C. ( 2017 ). The neurovascular unit coming of age: A journey through neurovascular coupling in health and disease . Neuron , 96 ( 1 ), 17 – 42 . 10.1016/j.neuron.2017.07.030 28957666 PMC5657612

[b35] Jerman , T. , Pernus , F. , Likar , B. , & Spiclin , Z. ( 2016 ). Enhancement of vascular structures in 3D and 2D angiographic images . IEEE Transactions on Medical Imaging , 35 ( 9 ), 2107 – 2118 . 10.1109/TMI.2016.2550102 27076353

[b36] Jochimsen , T. H. , Norris , D. G. , Mildner , T. , & Möller , H. E. ( 2004 ). Quantifying the intra- and extravascular contributions to spin-echo fMRI at 3 t . Magnetic Resonance in Medicine , 52 ( 4 ), 724 – 732 . 10.1002/mrm.20221 15389950

[b37] Kastrup , A. , Li , T.-Q. , Takahashi , A. , Glover , G. H. , & Moseley , M. E. ( 1998 ). Functional magnetic resonance imaging of regional cerebral blood oxygenation changes during breath holding . Stroke , 29 ( 12 ), 2641 – 2645 . 10.1161/01.STR.29.12.2641 9836778

[b38] Koopmans , P. J. , Barth , M. , & Norris , D. G. ( 2010 ). Layer-specific BOLD activation in human V1 . Human Brain Mapping , 31 ( 9 ), 1297 – 1304 . 10.1002/hbm.20936 20082333 PMC6870878

[b39] Ladd , M. E. , Quick , H. H. , Speck , O. , Bock , M. , Doerfler , A. , Forsting , M. , Hennig , J. , Ittermann , B. , Möller , H. E. , Nagel , A. M. , Niendorf , T. , Remy , S. , Schaeffter , T. , Scheffler , K. , Schlemmer , H.-P. , Schmitter , S. , Schreiber , L. , Shah , N. J. , Stöcker , T. , … Zaitsev , M . ( 2023 ). Germany’s journey toward 14 tesla human magnetic resonance . Magnetic Resonance Materials in Physics, Biology and Medicine , 36 ( 2 ), 191 – 210 . 10.1007/s10334-023-01085-z PMC10140098 37029886

[b40] Lai , S. , Hopkins , A. L. , Haacke , E. M. , Li , D. , Wasserman , B. A. , Buckley , P. , Friedman , L. , Meltzer , H. , Hedera , P. , & Friedland , R. ( 1993 ). Identification of vascular structures as a major source of signal contrast in high resolution 2D and 3D functional activation imaging of the motor cortex at 1.5T preliminary results . Magnetic Resonance in Medicine , 30 ( 3 ), 387 – 392 . 10.1002/mrm.1910300318 8412613

[b41] Linninger , A. , Hartung , G. , Badr , S. , & Morley , R. ( 2019 ). Mathematical synthesis of the cortical circulation for the whole mouse brain-part I. Theory and image integration . Computers in Biology and Medicine , 110 , 265 – 275 . 10.1016/j.compbiomed.2019.05.004 31247510

[b42] Lorthois , S. , Cassot , F. , & Lauwers , F. ( 2011 ). Simulation study of brain blood flow regulation by intra-cortical arterioles in an anatomically accurate large human vascular network: Part I: Methodology and baseline flow . NeuroImage , 54 ( 2 ), 1031 – 1042 . 10.1016/j.neuroimage.2010.09.032 20869450

[b43] Lu , H. , Golay , X. , Pekar , J. J. , & Van Zijl , P. C. ( 2003 ). Functional magnetic resonance imaging based on changes in vascular space occupancy . Magnetic Resonance in Medicine , 50 ( 2 ), 263 – 274 . 10.1002/mrm.10519 12876702

[b44] Lutti , A. , Thomas , D. L. , Hutton , C. , & Weiskopf , N. ( 2013 ). High-resolution functional MRI at 3 T: 3D/2D echo-planar imaging with optimized physiological noise correction: High-resolution fMRI at 3 T . Magnetic Resonance in Medicine , 69 ( 6 ), 1657 – 1664 . 10.1002/mrm.24398 22821858 PMC4495253

[b45] Mansfield , P. ( 1977 ). Multi-planar image formation using NMR spin echoes . Journal of Physics C: Solid State Physics , 10 ( 3 ), L55 – L58 . 10.1088/0022-3719/10/3/004

[b46] Miller , K. L. ( 2012 ). FMRI using balanced steady-state free precession (SSFP) . NeuroImage , 62 ( 2 ), 713 – 719 . 10.1016/J.NEUROIMAGE.2011.10.040 22036996 PMC3398389

[b47] Miller , K. L. , Smith , S. M. , Jezzard , P. , Wiggins , G. C. , & Wiggins , C. J. ( 2007 ). Signal and noise characteristics of SSFP FMRI: A comparison with GRE at multiple field strengths . NeuroImage , 37 ( 4 ), 1227 – 1236 . 10.1016/j.neuroimage.2007.06.024 17706432

[b48] Moeller , S. , Yacoub , E. , Olman , C. A. , Auerbach , E. , Strupp , J. , Harel , N. , & Uğurbil , K. ( 2010 ). Multiband multislice GE-EPI at 7 Tesla, with 16-fold acceleration using partial parallel imaging with application to high spatial and temporal whole-brain fMRI . Magnetic Resonance in Medicine , 63 ( 5 ), 1144 – 1153 . 10.1002/mrm.22361 20432285 PMC2906244

[b49] Ogawa , S. , Lee , T. M. , Kay , A. R. , & Tank , D. W. ( 1990 ). Brain magnetic resonance imaging with contrast dependent on blood oxygenation . Proceedings of the National Academy of Sciences of the United States of America , 87 ( 24 ), 9868 – 9872 . 10.1073/pnas.87.24.9868 2124706 PMC55275

[b50] Ogawa , S. , Menon , R. S. , Tank , D. W. , Kim , S. G. , Merkle , H. , Ellermann , J. M. , & Ugurbil , K. ( 1993 ). Functional brain mapping by blood oxygenation level-dependent contrast magnetic resonance imaging. A comparison of signal characteristics with a biophysical model . Biophysical Journal , 64 ( 3 ), 803 – 812 . 10.1016/S0006-3495(93)81441-3 8386018 PMC1262394

[b51] Pais-Roldán , P. , Yun , S. D. , Palomero-Gallagher , N. , & Shah , N. J. ( 2023 ). Cortical depth-dependent human fMRI of resting-state networks using EPIK . Frontiers in Neuroscience , 17 , 1151544 . 10.3389/fnins.2023.1151544 37274214 PMC10232833

[b52] Pauling , L. , & Coryell , C. D. ( 1936 ). The magnetic properties and structure of hemoglobin, oxyhemoglobin and carbonmonoxyhemoglobin . Proceedings of the National Academy of Sciences of the United States of America , 22 ( 4 ), 210 – 216 . 10.1073/pnas.22.4.210 16577697 PMC1076743

[b53] Peng , S.-L. , Ravi , H. , Sheng , M. , Thomas , B. P. , & Lu , H. ( 2017 ). Searching for a truly “iso-metabolic” gas challenge in physiological MRI . Journal of Cerebral Blood Flow & Metabolism , 37 ( 2 ), 715 – 725 . 10.1177/0271678X16638103 26980756 PMC5381460

[b54] Pérez-Rodas , M. , Pohmann , R. , Scheffler , K. , & Heule , R. ( 2021 ). Intravascular BOLD signal characterization of balanced SSFP experiments in human blood at high to ultrahigh fields . Magnetic Resonance in Medicine , 85 ( 4 ), 2055 – 2068 . 10.1002/mrm.28575 33140871

[b55] Pinto , J. , Bright , M. G. , Bulte , D. P. , & Figueiredo , P. ( 2021 ). Cerebrovascular reactivity mapping without gas challenges: A methodological guide . Frontiers in Physiology , 11 , 608475 . 10.3389/fphys.2020.608475 33536935 PMC7848198

[b56] Pohmann , R. , Speck , O. , & Scheffler , K. ( 2016 ). Signal-to-noise ratio and MR tissue parameters in human brain imaging at 3, 7, and 9.4 Tesla using current receive coil arrays . Magnetic Resonance in Medicine , 75 ( 2 ), 801 – 809 . 10.1002/mrm.25677 25820458

[b57] Polimeni , J. R. , Fischl , B. , Greve , D. N. , & Wald , L. L. ( 2010 ). Laminar analysis of 7T BOLD using an imposed spatial activation pattern in human V1 . NeuroImage , 52 ( 4 ), 1334 – 1346 . 10.1016/j.neuroimage.2010.05.005 20460157 PMC3130346

[b58] Power , J. D. , Mitra , A. , Laumann , T. O. , Snyder , A. Z. , Schlaggar , B. L. , & Petersen , S. E. ( 2014 ). Methods to detect, characterize, and remove motion artifact in resting state fMRI . NeuroImage , 84 , 320 – 341 . 10.1016/j.neuroimage.2013.08.048 23994314 PMC3849338

[b59] Raichle , M. E. , & Mintun , M. A. ( 2006 ). Brain work and brain imaging . Annual Review of Neuroscience , 29 , 449 – 476 . 10.1146/annurev.neuro.29.051605.112819 16776593

[b60] Ress , D. , Glover , G. H. , Liu , J. , & Wandell , B. ( 2007 ). Laminar profiles of functional activity in the human brain . NeuroImage , 34 ( 1 ), 74 – 84 . 10.1016/j.neuroimage.2006.08.020 17011213

[b61] Santiesteban , F. M. M. , Swanson , S. D. , Noll , D. C. , & Anderson , D. J. ( 2006 ). Object orientation independence of susceptibility weighted imaging by using a sigmoid-type phase window . https://cds.ismrm.org/protected/06MProceedings/PDFfiles/02399.pdf

[b62] Scheffler , K. , & Ehses , P. ( 2016 ). High-resolution mapping of neuronal activation with balanced SSFP at 9.4 Tesla . Magnetic Resonance in Medicine , 76 ( 1 ), 163 – 171 . 10.1002/mrm.25890 26302451

[b63] Scheffler , K. , & Hennig , J. ( 2003 ). Is TrueFISP a gradient-echo or a spin-echo sequence? Magnetic Resonance in Medicine , 49 ( 2 ), 395 – 397 . 10.1002/mrm.10351 12541263

[b64] Scheffler , K. , Heule , R. , Báez-Yánez , M. G. , Kardatzki , B. , & Lohmann , G. ( 2019 ). The BOLD sensitivity of rapid steady-state sequences . Magnetic Resonance in Medicine , 81 ( 4 ), 2526 – 2535 . 10.1002/mrm.27585 30488986

[b65] Scheffler , K. , Seifritz , E. , Bilecen , D. , Venkatesan , R. , Hennig , J. , Deimling , M. , & Haacke , E. M. ( 2001 ). Detection of BOLD changes by means of a frequency-sensitive trueFISP technique: Preliminary results . NMR in Biomedicine , 14 ( 7 ), 490 – 496 . 10.1002/nbm.726 11746942

[b66] Setsompop , K. , Gagoski , B. A. , Polimeni , J. R. , Witzel , T. , Wedeen , V. J. , & Wald , L. L. ( 2012 ). Blipped-controlled aliasing in parallel imaging for simultaneous multislice echo planar imaging with reduced *g* -factor penalty . Magnetic Resonance in Medicine , 67 ( 5 ), 1210 – 1224 . 10.1002/mrm.23097 21858868 PMC3323676

[b67] Shajan , G. , Kozlov , M. , Hoffmann , J. , Turner , R. , Scheffler , K. , & Pohmann , R. ( 2014 ). A 16-channel dual-row transmit array in combination with a 31-element receive array for human brain imaging at 9.4 T . Magnetic Resonance in Medicine , 71 ( 2 ), 870 – 879 . 10.1002/mrm.24726 23483645

[b68] Siero , J. C. , Petridou , N. , Hoogduin , H. , Luijten , P. R. , & Ramsey , N. F. ( 2011 ). Cortical depth-dependent temporal dynamics of the BOLD response in the human brain . Journal of Cerebral Blood Flow and Metabolism , 31 ( 10 ), 1999 – 2008 . 10.1038/jcbfm.2011.57 21505479 PMC3208150

[b69] Smith , S. M. , Jenkinson , M. , Woolrich , M. W. , Beckmann , C. F. , Behrens , T. E. , Johansen-Berg , H. , Bannister , P. R. , De Luca , M. , Drobnjak , I. , Flitney , D. E. , Niazy , R. K. , Saunders , J. , Vickers , J. , Zhang , Y. , De Stefano , N. , Brady , J. M. , & Matthews , P. M. ( 2004 ). Advances in functional and structural MR image analysis and implementation as FSL . NeuroImage , 23 , S208 – S219 . 10.1016/j.neuroimage.2004.07.051 15501092

[b70] Song , A. W. , Wong , E. C. , Tan , S. G. , & Hyde , J. S. ( 1996 ). Diffusion weighted fMRI at 1.5 t . Magnetic Resonance in Medicine , 35 ( 2 ), 155 – 158 . 10.1002/mrm.1910350204 8622577

[b71] Speck , O. , Stadler , J. , & Zaitsev , M. ( 2008 ). High resolution single-shot EPI at 7T . Magnetic Resonance Materials in Physics, Biology and Medicine , 21 ( 1 ), 73 – 86 . 10.1007/s10334-007-0087-x 17973132

[b72] Stehling , M. K. , Schmitt , F. , & Ladebeck , R. ( 1993 ). Echo-planar MR imaging of human brain oxygenation changes . Journal of Magnetic Resonance Imaging , 3 ( 3 ), 471 – 474 . 10.1002/jmri.1880030307 8324305

[b73] Stirnberg , R. , & Stöcker , T. ( 2021 ). Segmented k-space blipped-controlled aliasing in parallel imaging for high spatiotemporal resolution EPI . Magnetic Resonance in Medicine , 85 ( 3 ), 1540 – 1551 . 10.1002/mrm.28486 32936488

[b74] Triantafyllou , C. , Hoge , R. , Krueger , G. , Wiggins , C. , Potthast , A. , Wiggins , G. , & Wald , L. ( 2005 ). Comparison of physiological noise at 1.5 T, 3 T and 7 T and optimization of fMRI acquisition parameters . NeuroImage , 26 ( 1 ), 243 – 250 . 10.1016/j.neuroimage.2005.01.007 15862224

[b75] Turner , R. ( 2002 ). How much codex can a vein drain? Downstream dilution of activation-related cerebral blood oxygenation changes . NeuroImage , 16 ( 4 ), 1062 – 1067 . 10.1006/nimg.2002.1082 12202093

[b76] Uǧurbil , K. , Hu , X. , Chen , W. , Zhu , X. H. , Kim , S. G. , & Georgopoulos , A. ( 1999 ). Functional mapping in the human brain using high magnetic fields . Philosophical Transactions of the Royal Society B: Biological Sciences , 354 ( 1387 ), 1195 – 1213 . 10.1098/rstb.1999.0474 PMC1692632 10466146

[b77] Uludaǧ , K. , Müller-Bierl , B. , & Uǧurbil , K. ( 2009 ). An integrative model for neuronal activity-induced signal changes for gradient and spin echo functional imaging . NeuroImage , 48 ( 1 ), 150 – 165 . 10.1016/j.neuroimage.2009.05.051 19481163

[b78] Vaculčiaková , L. , Podranski , K. , Edwards , L. J. , Ocal , D. , Veale , T. , Fox , N. C. , Haak , R. , Ehses , P. , Callaghan , M. F. , Pine , K. J. , & Weiskopf , N. ( 2022 ). Combining navigator and optical prospective motion correction for high-quality 500 μ m resolution quantitative multi-parameter mapping at 7T . Magnetic Resonance in Medicine , 88 ( 2 ), 787 – 801 . 10.1002/mrm.29253 35405027

[b79] Viessmann , O. , Scheffler , K. , Bianciardi , M. , Wald , L. L. , & Polimeni , J. R. ( 2019 ). Dependence of resting-state fMRI fluctuation amplitudes on cerebral cortical orientation relative to the direction of B0 and anatomical axes . NeuroImage , 196 , 337 – 350 . 10.1016/j.neuroimage.2019.04.036 31002965 PMC6559854

[b80] Walsh , D. O. , Gmitro , A. F. , & Marcellin , M. W. ( 2000 ). Adaptive reconstruction of phased array MR imagery . Magnetic Resonance in Medicine , 43 ( 5 ), 682 – 690 . 10.1002/(sici)1522-2594(200005)43:5<682::aid-mrm10>3.0.co;2-g 10800033

[b81] Weisskoff , R. , Zuo , C. S. , Boxerman , J. L. , & Rosen , B. R. ( 1994 ). Microscopic susceptibility variation and transverse relaxation: Theory and experiment . Magnetic Resonance in Medicine , 31 ( 6 ), 601 – 610 . 10.1002/mrm.1910310605 8057812

[b82] Yacoub , E. , Shmuel , A. , Pfeuffer , J. , Van De Moortele , P.-F. , Adriany , G. , Andersen , P. , Vaughan , J. T. , Merkle , H. , Ugurbil , K. , & Hu , X. ( 2001 ). Imaging brain function in humans at 7 Tesla . Magnetic Resonance in Medicine , 45 ( 4 ), 588 – 594 . 10.1002/mrm.1080 11283986

[b83] Zhong , K. , Leupold , J. , Hennig , J. , & Speck , O. ( 2006 ). fMRI activation in human visual cortex measured with steady state free precession at 3 Tesla . In 14th ISMRM, Seattle (Vol. 14 , p. 3296 ). https://cds.ismrm.org/protected/06MProceedings/PDFfiles/03296.pdf

[b84] Zhong , X. Z. , Polimeni , J. R. , & Chen , J. J. ( 2024 ). Predicting the macrovascular contribution to resting-state fMRI functional connectivity at 3 Tesla: A model-informed approach . bioRxiv . 10.1101/2024.02.13.580143 PMC12290778 40800337

