# Supplementary material for: Macrovascular contributions to resting-state fMRI signals: A comparison between EPI and bSSFP at 9.4 Tesla
Source: Imaging Neurosci (Camb). 2025 Jan 7;3:imag_a_00435. doi: 10.1162/imag_a_00435 (PMC12320002; doi:10.1162/imag_a_00435)
Supplement: Supplementary Material [file imag_a_00435-supp.pdf]

Macrovascular contributions to resting-state fMRI signals: A comparison between EPI and bSSFP at 9.4 Tesla

Dana Ramadan[1,*], Sebastian Mueller[1], Ruediger Stirnberg[2], Dario Bosch[1,3], Philipp Ehses[2], Klaus Scheffler[1,3], and Jonas Bause[1]

[1]High-Field Magnetic Resonance Center, Max Planck Institute for Biological Cybernetics, Tuebingen, Germany
[2]German Center for Neurodegenerative Diseases (DZNE), Bonn, Germany
[3]Department of Biomedical Magnetic Resonance, University of Tuebingen, Tuebingen, Germany
[*]Correspondence: dana.ramadan@tuebingen.mpg.de

Supplemental Material

Table S1: Relative CV values plotted in Figure 3 using 15 bins with the same amount of voxels in each bin. Corresponding mean θ_{B_0} values are not shown here, but can be seen in the plots. R: entire cortical ribbon; D1-5: different cortical depths.

**3D bSSFP**															
R	3.53	2.88	1.71	0.57	0.61	-0.39	-0.97	-1.08	-1.29	-1.38	-0.82	-1.06	-0.62	-0.54	0.00
D1	0.19	-1.16	-2.72	-3.36	-2.11	-3.22	-3.79	-2.32	-2.63	-2.86	-2.23	-0.84	-1.08	-0.20	0.00
D2	1.35	0.93	0.20	-1.22	-1.18	-1.31	-1.68	-1.75	-1.46	-2.25	-1.05	-1.30	-0.88	-0.62	0.00
D3	1.28	0.08	-0.53	-1.09	-1.27	-1.25	-2.15	-2.11	-2.28	-2.08	-1.44	-2.06	-1.55	-2.06	0.00
D4	6.43	5.12	3.37	2.43	1.51	0.10	-0.16	-0.57	-0.97	-1.02	-0.59	-0.74	-0.32	-0.10	0.00
D5	7.16	6.86	4.73	3.52	3.24	1.70	1.26	0.23	-0.04	-0.07	0.21	-0.10	-0.12	0.00	0.00
**3D EPI**															
R	43.52	34.50	23.21	17.66	12.66	6.21	2.85	0.94	-0.77	0.45	-0.44	-1.39	-0.07	0.35	0.00
D1	53.59	48.36	35.49	25.88	21.06	3.35	8.35	0.33	1.75	0.96	0.81	-0.57	-0.85	-0.30	0.00
D2	49.97	37.83	25.70	18.30	13.54	5.10	2.48	0.01	-1.52	-1.14	-2.49	-3.02	-0.70	-0.54	0.00
D3	42.52	32.19	19.82	17.45	11.70	8.24	4.89	2.89	1.03	0.73	1.01	1.19	2.43	2.42	0.00
D4	33.49	28.12	20.64	15.65	10.83	7.36	5.01	3.13	1.15	1.19	0.45	-0.14	0.45	0.34	0.00
D5	29.16	29.95	20.85	16.75	9.27	6.64	4.09	2.71	2.04	1.51	0.46	0.87	0.34	-0.56	0.00
**2D EPI**															
R	23.98	15.88	10.48	5.71	3.62	1.39	0.39	-1.05	-0.87	-1.09	-0.67	-0.28	-0.02	-0.31	0.00
D1	53.06	38.17	24.72	14.03	9.88	5.54	3.70	0.81	0.41	1.19	1.38	0.63	0.74	0.72	0.00
D2	29.57	19.35	12.49	7.70	3.78	1.39	0.35	-0.98	-0.41	-0.95	-1.53	-0.44	0.56	-0.44	0.00
D3	12.81	8.79	4.83	1.63	0.80	-0.55	-1.01	-1.52	-1.27	-2.04	-1.66	-0.40	-0.52	-0.72	0.00
D4	11.17	7.02	4.60	2.24	1.13	-0.27	-1.42	-2.31	-2.08	-1.94	-1.50	-0.65	-0.27	-0.29	0.00
D5	12.12	9.80	6.68	3.70	2.50	0.67	0.73	-0.83	-0.81	-1.06	-0.92	0.59	-0.33	-0.08	0.00

Up-sampling before depths segmentation in LayNii

Up-sampling is recommended before performing depth segmentation using LayNii (Huber et al., 2021) to obtain smoother equi-distant results. Moreover, LayNii works best with straight patches and performs poorer in curved regions. Therefore, we up-sampled the co-registered cortical ribbon by a factor of four to get voxel dimensions of $0.275\,\mathrm{mm}$ isotropic and be able to divide the ribbon into five equi-distant depths with Depth 1 (D1) and Depth 5 (D5) closest to CSF and WM, respectively. The difference between the original data and the up-sampled data using nearest neighbor interpolation can be seen in Figure S1 that shows the number of voxels per cortical depth without (A) and with (B) up-sampling.

Note that every third slice in each dimension after up-sampling is considered, as otherwise data points would be repetitive. An uneven distribution of voxels (with more voxels in the middle layers) can be seen for the original data without up-sampling, both from the bar plots and from the overlaid depth profiles displayed below. Therefore, unless otherwise stated, all results shown are obtained after up-sampling.

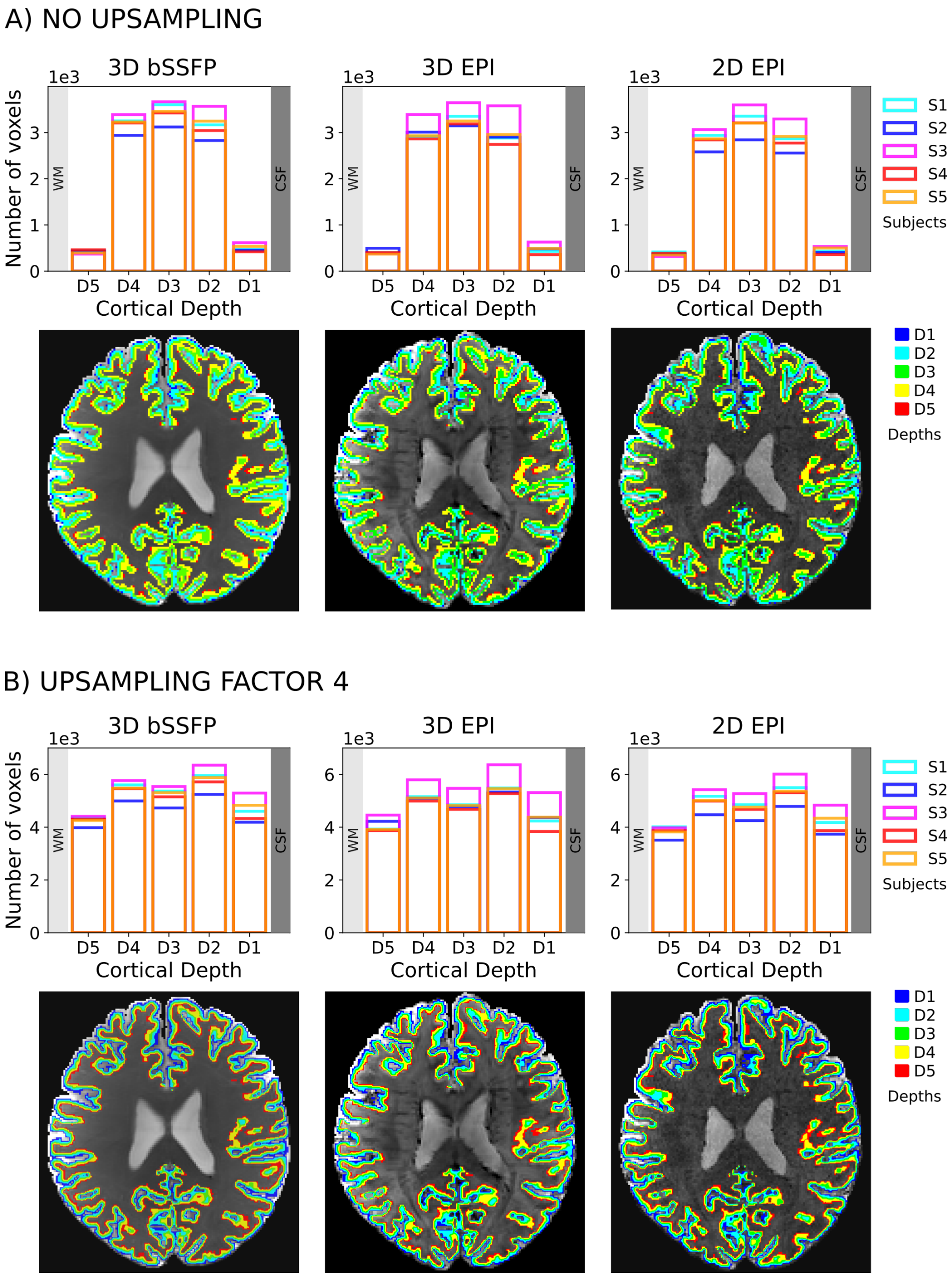

Figure S1: *Distribution of voxels in the cortical depths* without (A) and with (B) up-sampling by a factor of four. Bar plots of the voxel counts are shown for all subjects (upper row) and the depths calculated with LayNii are overlaid on the mean functional image of one subject (lower row) for all sequences. The depths are more equally distributed after up-sampling.

Removing the orientation bias

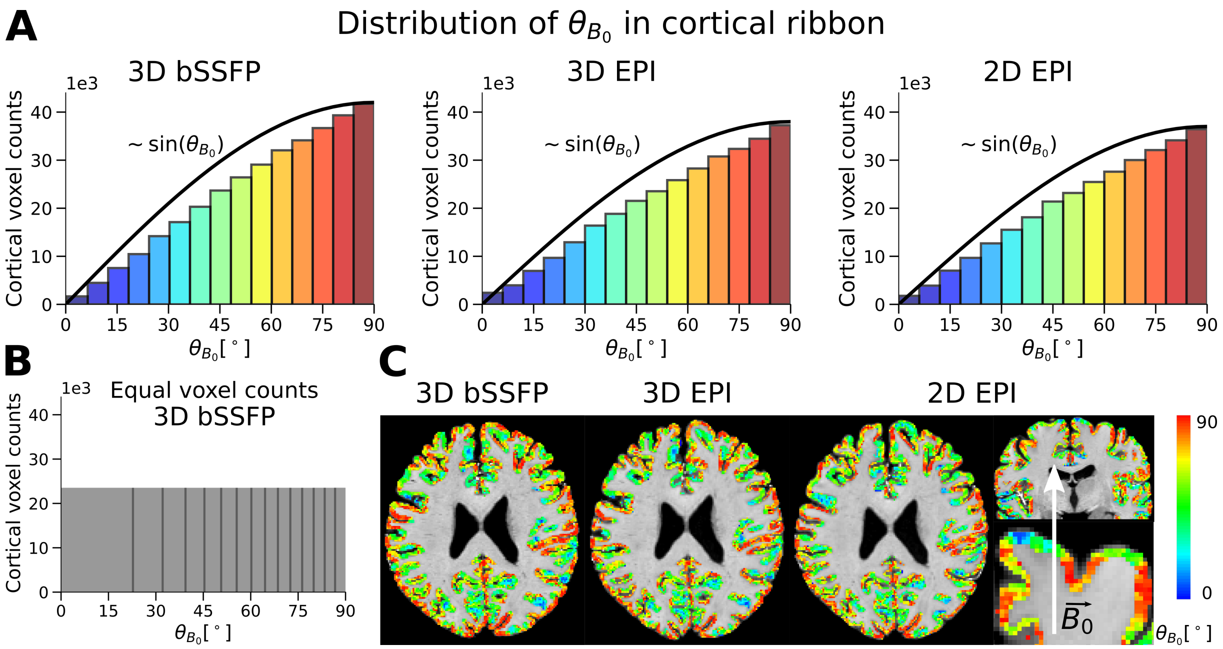

Figure S2: *Distribution of* θ_{B_0} in the entire cortical ribbon after co-registration (and warping) (A). It can be seen that the distribution follows a sine curve. In order to perform the analysis without a higher weighting of voxels around 90°, the orientation intervals are adjusted as shown in (B) for further analysis. The co-registration (and warping) results for the orientation values for all three sequences are visualized (C). In addition to the axial slice (through-plane $\overrightarrow{B_0}$ vector) a coronal slice and a zoomed view for the 2D EPI sequence is shown with the $\overrightarrow{B_0}$ vector indicated as a white arrow.

Mean and standard deviation on the cortical orientation

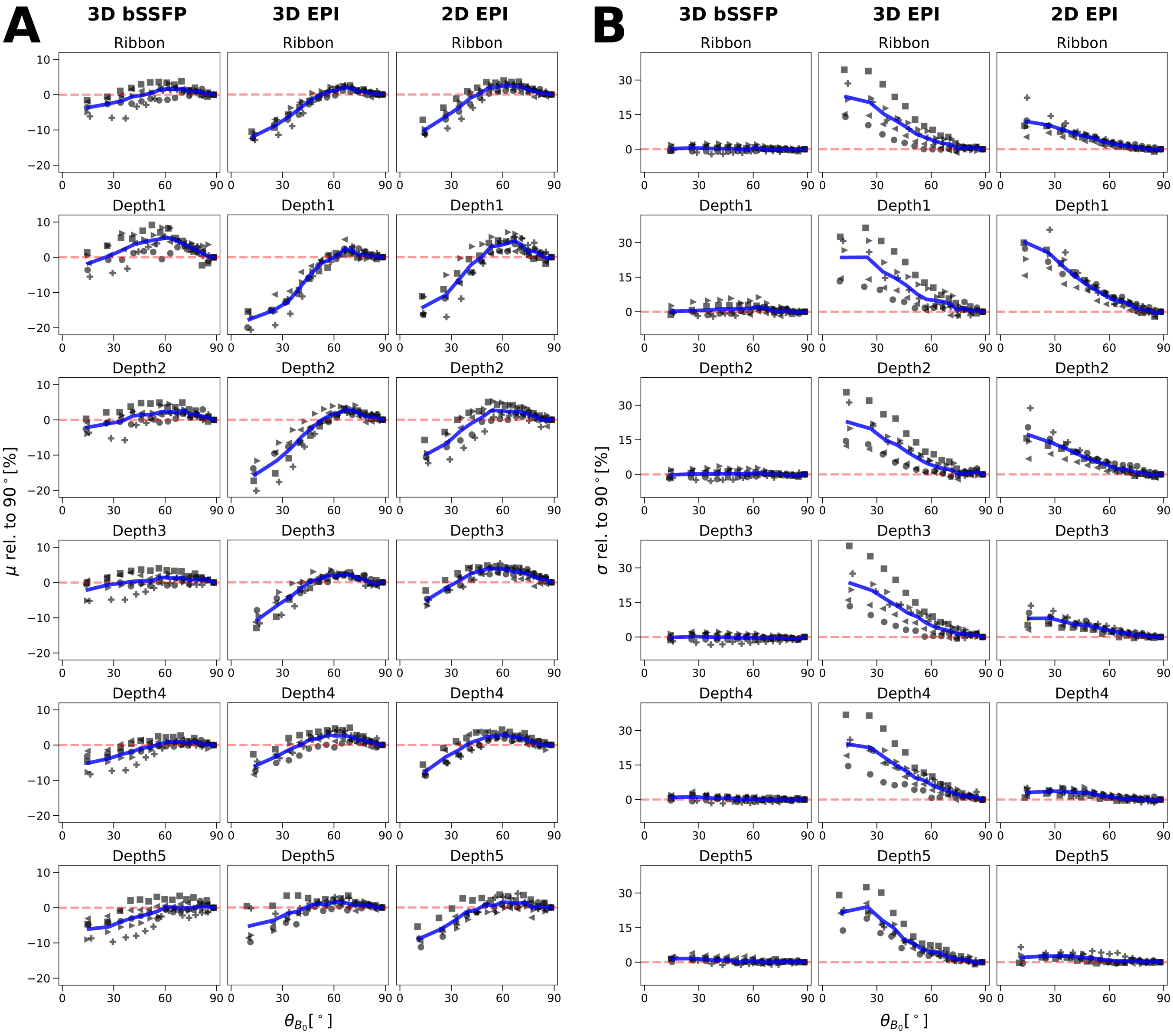

Figure S3: Mean **(A)** and standard deviation **(B)** of the time series plotted against the cortical orientation relative to B0. For details, see Figure 3.

Results of session 2

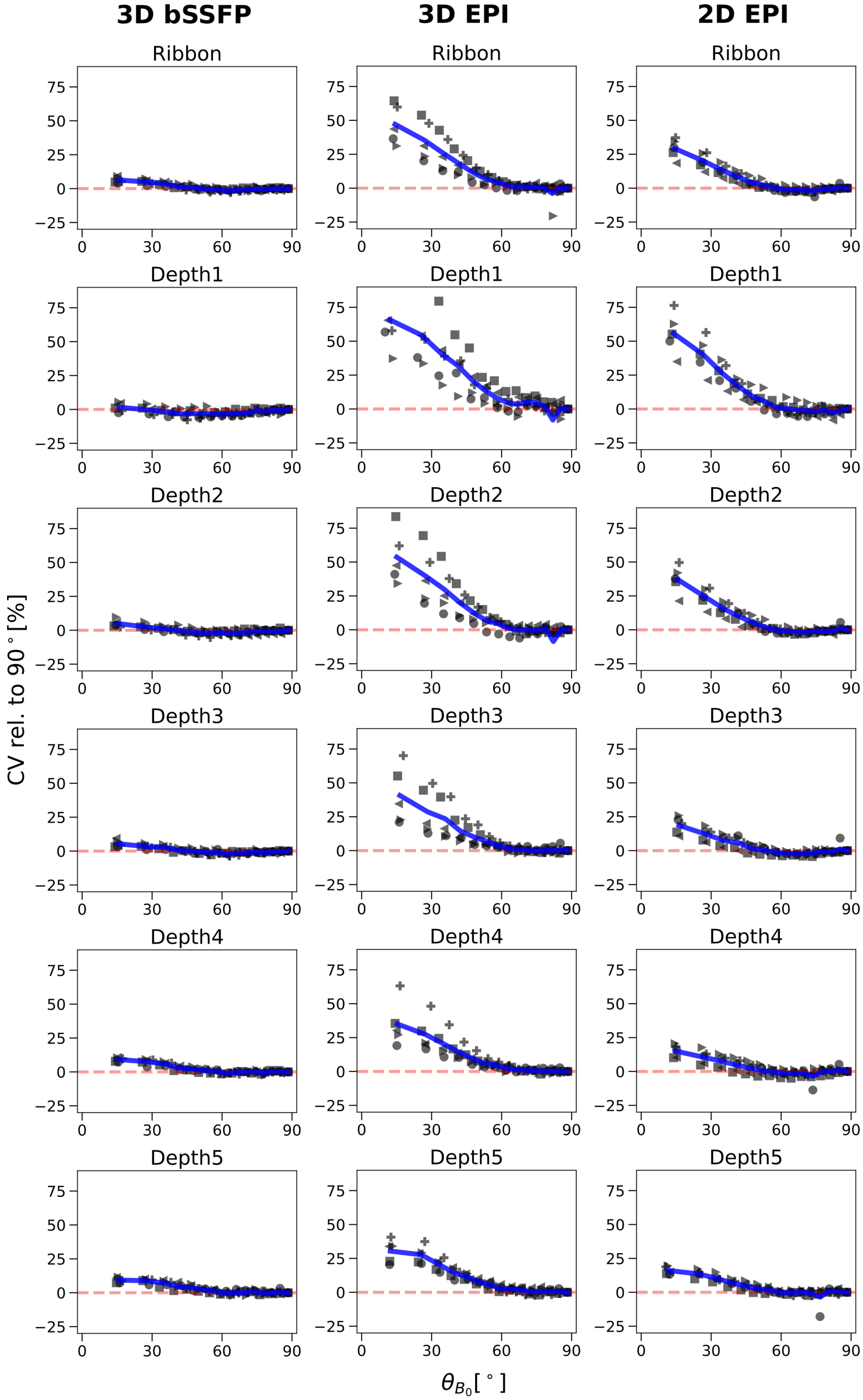

Figure S4: *CV_{rel} plotted against the cortical orientation to B0 in session 2.* Mean CV_{rel} values from one run (3D bSSFP and 3D EPI) and four runs (2D EPI) of all five subjects are shown in blue. See Figure 3 for details.

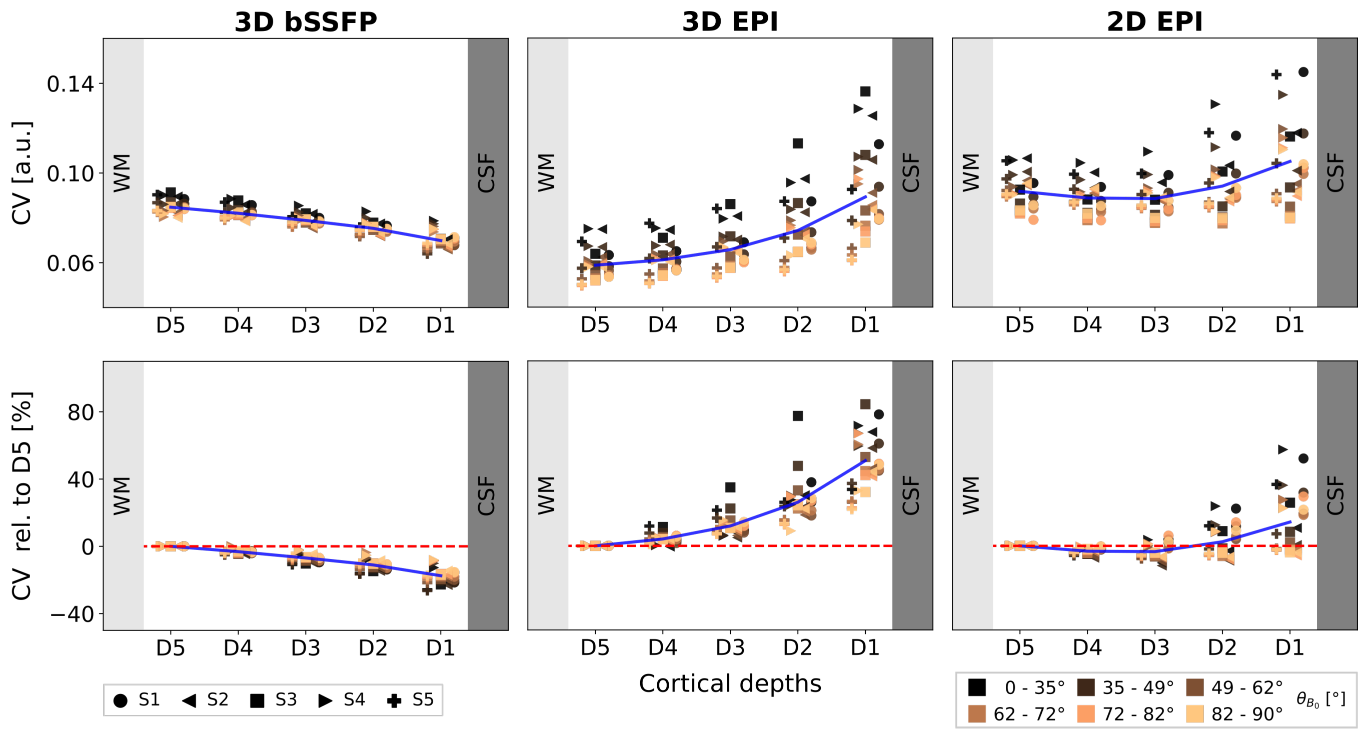

Figure S5: *CV plotted against the cortical depths in session 2* (D1 and D5 closest to CSF and WM, respectively). Mean CV_{rel} values from one run (3D bSSFP and 3D EPI) and four runs (2D EPI) of all five subjects are shown in blue. See Figure 4 for details.

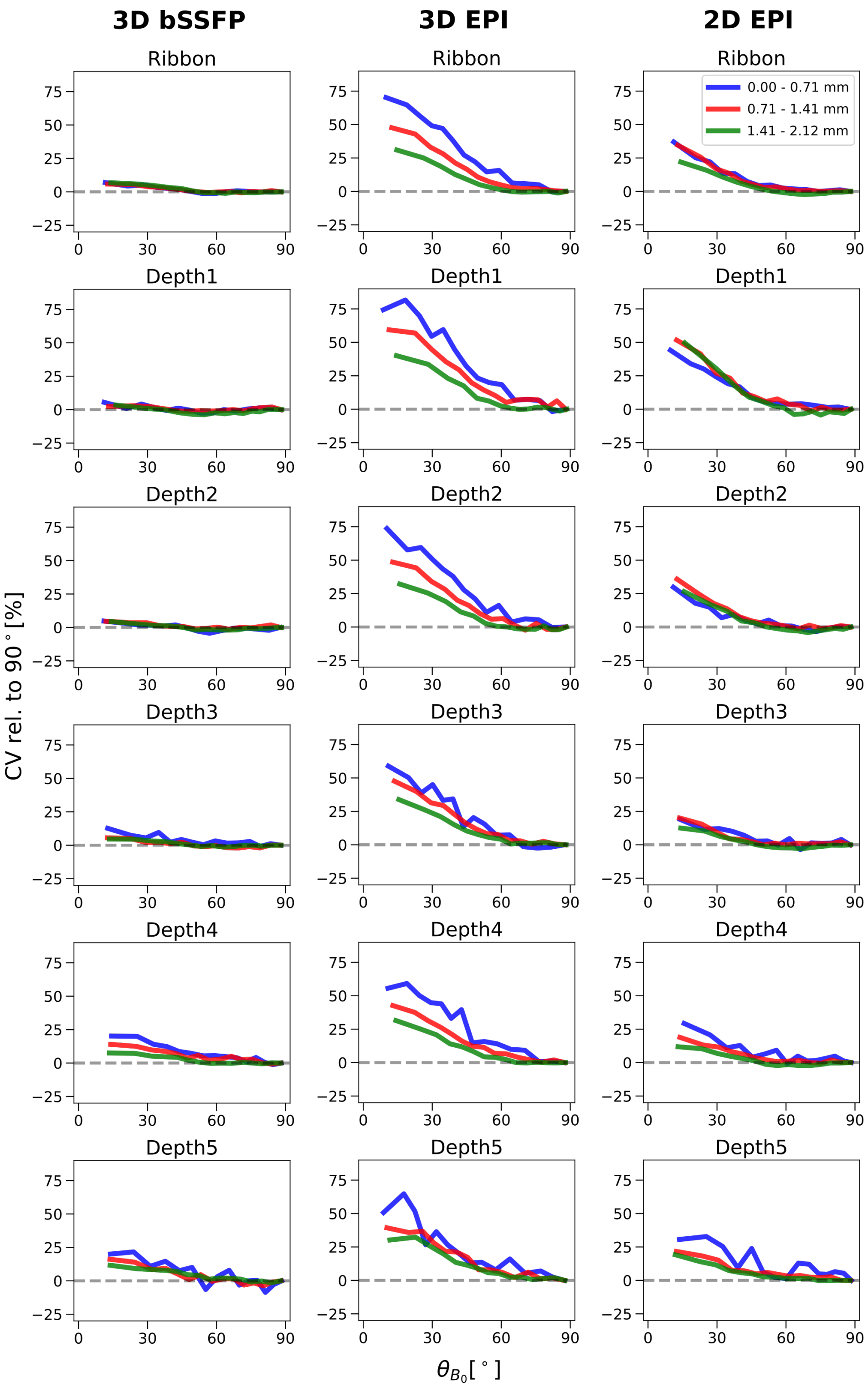

Figure S6: *CV_{rel} plotted against the cortical orientation to B0 in session 2.* Mean CV_{rel} values from one run (3D bSSFP and 3D EPI) and four runs (2D EPI) of four subjects are shown. Voxels are binarized into three pools depending on their distance from veins. The blue, red and green lines correspond to voxels in high ($0\,\mathrm{mm}$ to $0.71\,\mathrm{mm}$), medium ($0.71\,\mathrm{mm}$ to $1.41\,\mathrm{mm}$) and low ($1.41\,\mathrm{mm}$ to $2.12\,\mathrm{mm}$) proximity to the veins. See Figure 5C for details.

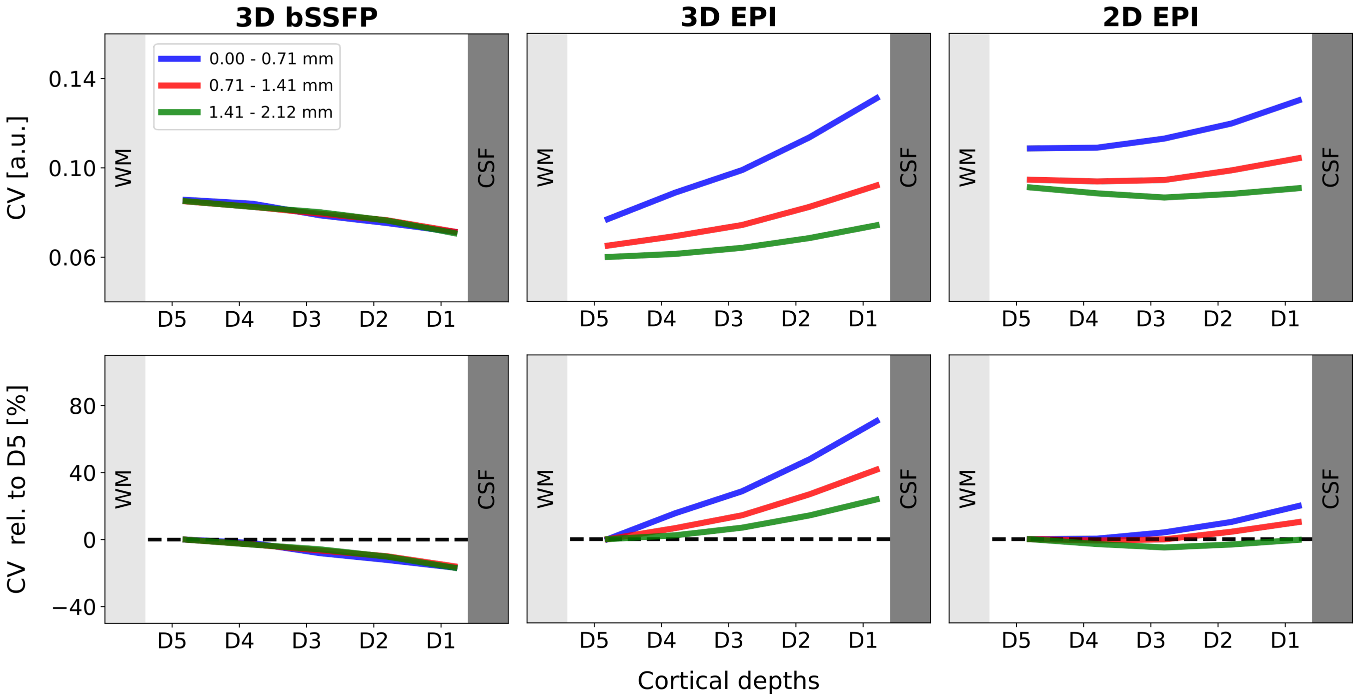

Figure S7: *CV plotted against the cortical depths in session 2.* Mean CV values from one run (3D bSSFP and 3D EPI) and four runs (2D EPI) of four subjects are shown. Voxels are binarized into three pools depending on their distance to veins. The blue, red and green lines correspond to voxels in high ($0\,\mathrm{mm}$ to $0.71\,\mathrm{mm}$), medium ($0.71\,\mathrm{mm}$ to $1.41\,\mathrm{mm}$) and low ($1.41\,\mathrm{mm}$ to $2.12\,\mathrm{mm}$) proximity to the veins. See Figure 5B for details.

Effect of voxel size

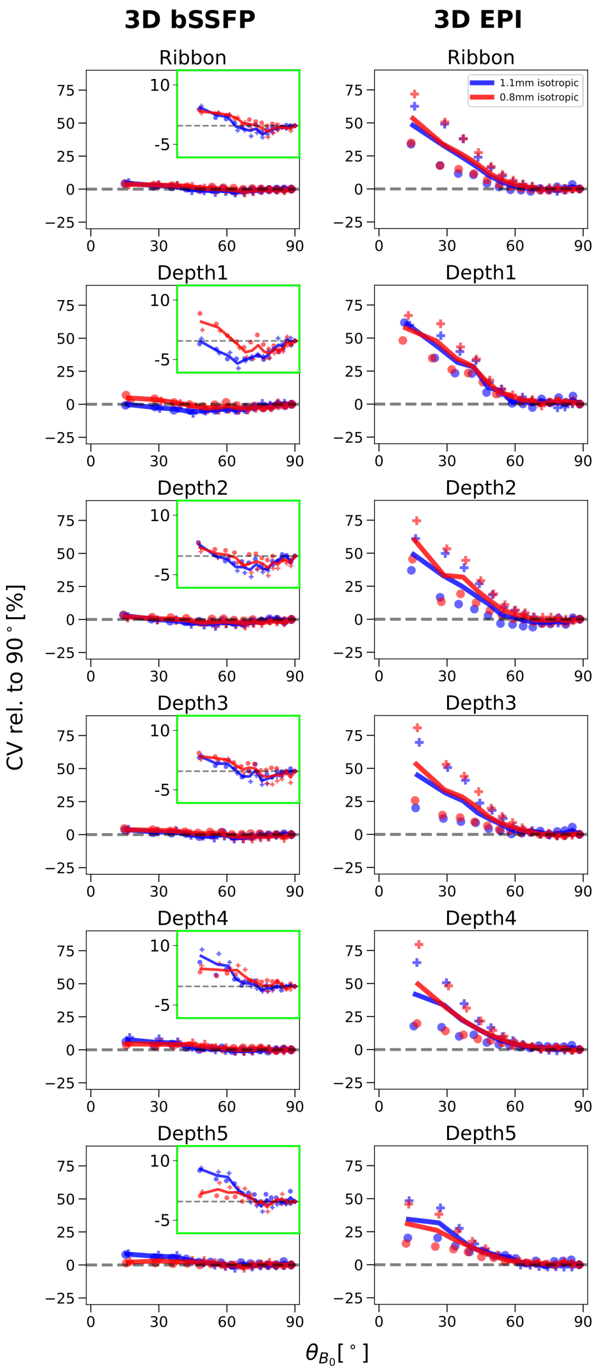

Figure S8: *CV_{rel} plotted against the cortical orientation to B0:* Mean CV_{rel} values from one run of two subjects (S1 and S5) from the same session are shown. Curves from data with voxel sizes of $1.1\,\mathrm{mm}$ and $0.8\,\mathrm{mm}$ isotropic are shown in blue and red, respectively. The acquisition parameters used for the high resolution images were as follows: **3D bSSFP:** nominal matrix size: 238x238x48, number of volumes: 92, resolution: 0.8 mm isotropic, TR/TE = 3.57 ms/1.77 ms, TRvol = 4090 ms, nominal FA = 11○, slice pF = 5/8, GRAPPA = 5x1, RO bandwidth = 1050 Hz/px. **3D EPI**: nominal matrix size: 238x238x58, number of volumes: 92, resolution: 0.8 mm isotropic, TR_{vol} = 3500 ms, TE = 17 ms, FA = 15○, phase pF = 6/8, CAIPIRINHA = 2x2, PE1 = anterior-posterior (AP), PE2 = HF, interleaved multi-shot segmentation, factor = 3, nominal echo spacing = 1.09 ms, RO bandwidth = 1106 Hz/px.

Effect of RETROICOR and temporal smoothing

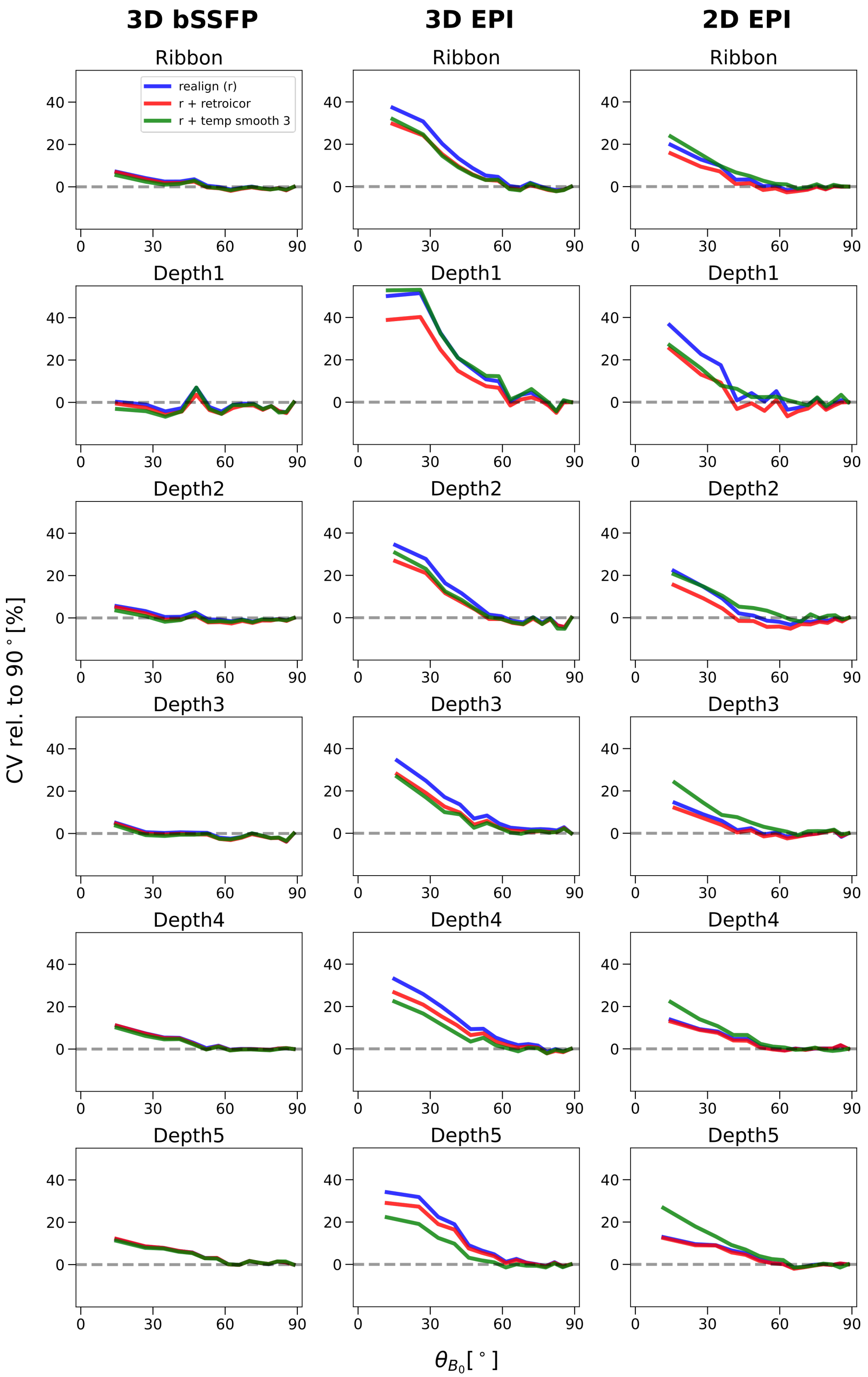

Figure S9: *Comparison of cortical orientation dependence after physiological noise regression and temporal smoothing:* CV_{rel} plotted against θ_{B_0} for 3D bSSFP (left), 3D EPI (middle) and 2D EPI (right). The average CV_{rel} calculated after motion correction only [realign] **(blue)**, realignment and physiological noise regression using RETROICOR [r + retroicor] **(red)**, and realignment and temporal smoothing of the data with a moving average of 3 TR_{vol} [r + temp smooth 3] **(green)** are plotted against θ_{B_0}. One subject (S4) is shown here, because the acquisition of the physiological parameters was only reliable for all runs of this subject. For more details, see caption of Figure 3.

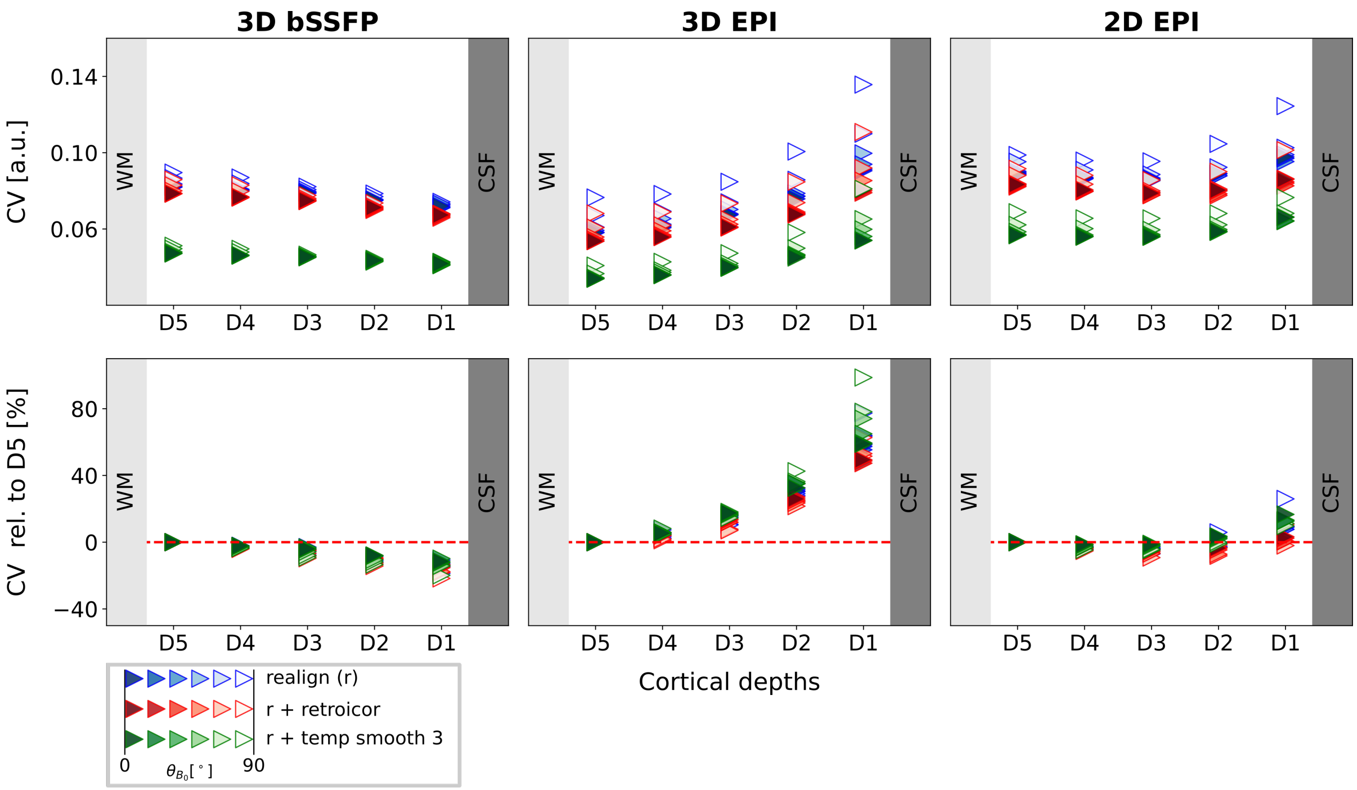

Figure S10: *Comparison of cortical orientation dependence after physiological noise correction using RETROICOR (red) and temporal smoothing with a moving average of 3 TR_{vol}:* The CV was plotted against the cortical depths for 3D bSSFP (left), 3D EPI (middle) and 2D EPI (right). The absolute (top) CV values and relative to D5 (bottom) are shown. Each plotted point represents the mean of all CV values within a specific range of θ_{B_0} values. Six ranges with equal voxel counts were plotted for each subject, with lighter colors corresponding to higher θ_{B_0} values around 90°. Only one subject (S4) is shown here, because the acquisition of the physiological parameters was only reliable for all runs of this subject.

Fitting the cortical orientation dependence

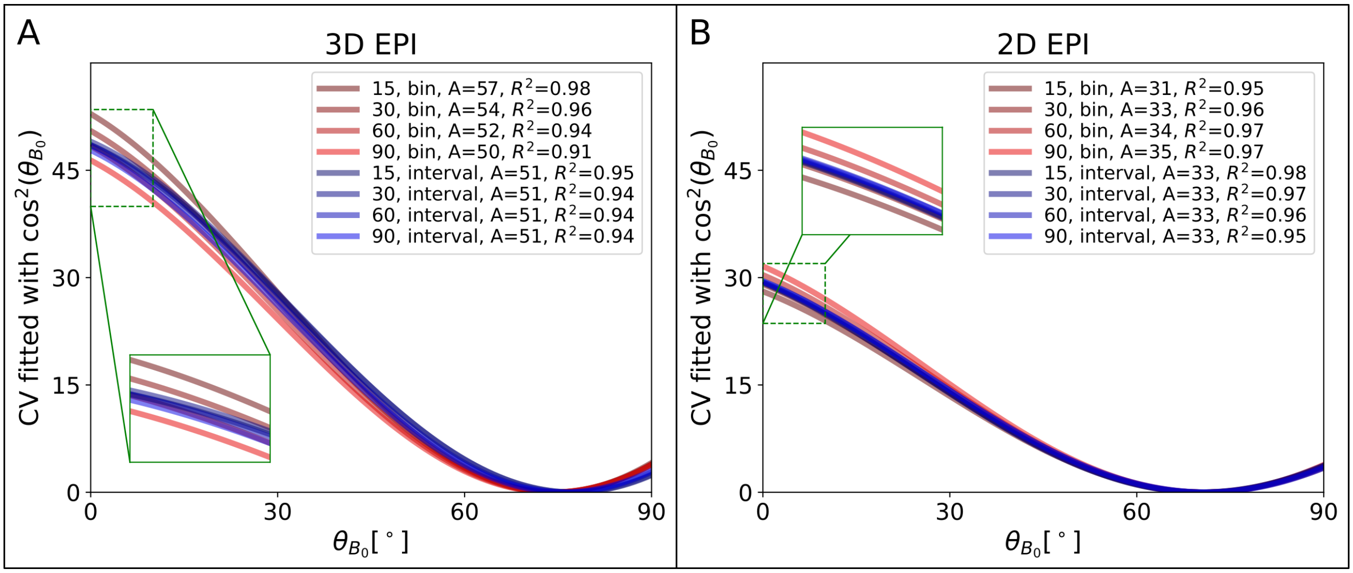

Figure S11: *Fitting of cortical orientation dependence*. Using data points plotted in the cortical ribbon for both 2D and 3D EPI, we fitted $CV = A\cos^2(\theta_{B_0} + C)$ function to eight different plotting methods of the cortical data. The major differences between the plotted function are the following: Data were plotted with constant bin size (as in this work) and with constant interval size (which would have the distribution bias of the cortical angles), shown in red and blue tones, respectively. The second variable we changed was the number of bins plotted before the fitting of the data, which were in the range of [15,30,60,90] bins, given by the hue of the plotted fits. The legend shows these parameters, the amplitude of the $\cos^2(\theta_{B_0})$ function (A), and the quality of the fit (R^2). The green box shows a zoomed-in version in the range of $\theta_{B_0} = 0^\circ - 10^\circ$.

Effect of motion regression

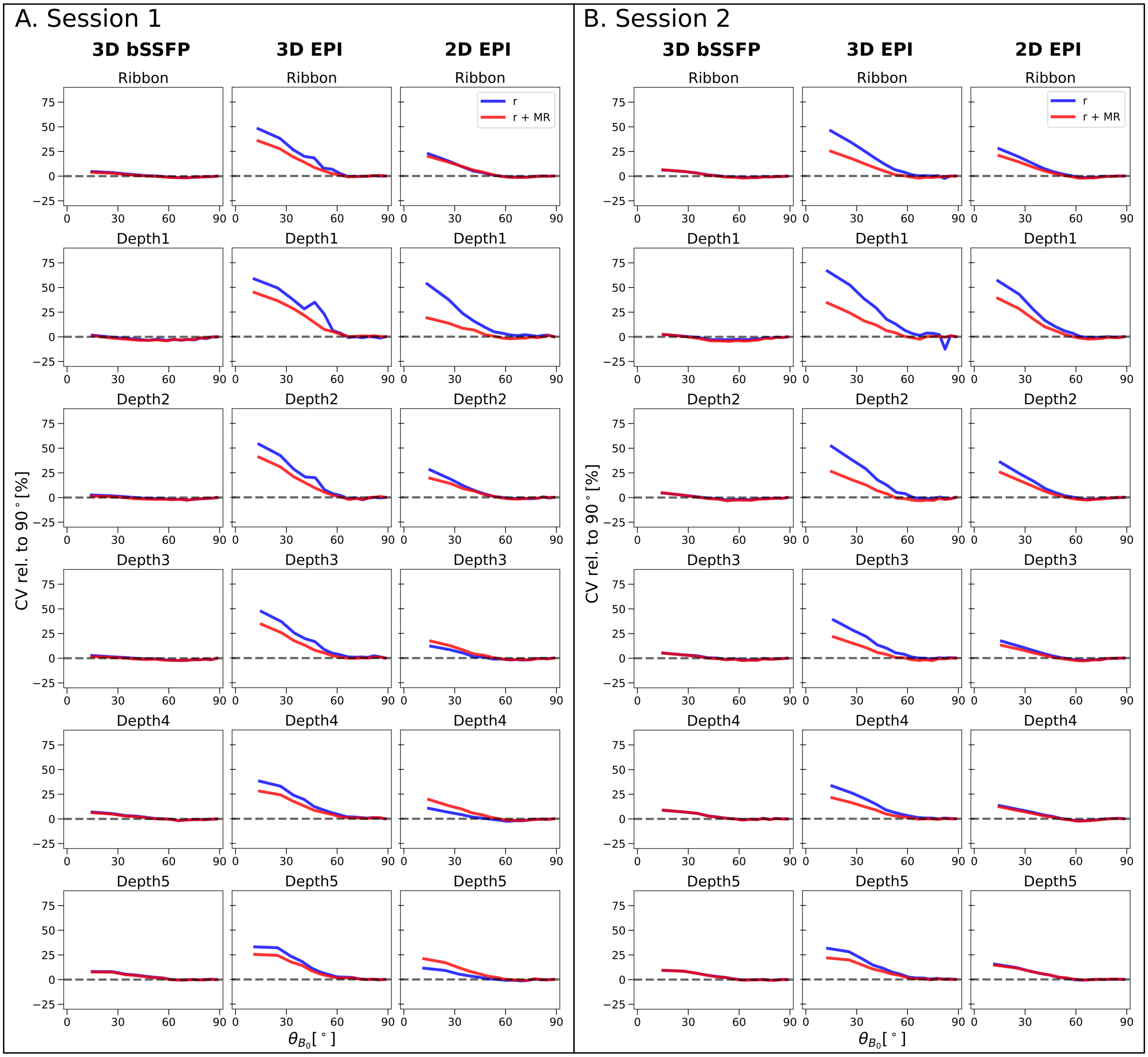

Figure S12: *Comparison of cortical orientation dependence after motion regression:* CV_{rel} plotted against θ_{B_0} for 3D bSSFP (left), 3D EPI (middle) and 2D EPI (right). The average CV_{rel} calculated after motion correction only [r] **(blue)** and realignment and motion regression of the data [r + MR] **(red)** are plotted against θ_{B_0}. One run of all subjects is shown here. For more details, see caption of Figure 3.
